# Senotherapy for chronic lung disease

**DOI:** 10.1016/j.pharmr.2025.100069

**Published:** 2025-05-28

**Authors:** Peter J. Barnes

**Affiliations:** National Heart and Lung Institute, Imperial College, London, United Kingdom

## Abstract

Chronic respiratory diseases are an enormous burden on healthcare and the third ranked cause of death globally. There is now compelling evidence that acceleration of lung aging and associated cellular senescence is a key driving mechanism of several chronic lung diseases, particularly chronic obstructive pulmonary disease and idiopathic pulmonary fibrosis. Senescent cells, arising from oxidative stress and unrepaired damage, can accumulate in the lung and develop a senescence-associated secretory phenotype, spreading senescence and resulting in disease progression. In addition, there is a reduction in normally protective antiaging molecules, such as sirtuins, in the lungs. The role of cellular senescence in chronic lung disease has driven interest in senotherapy that targets senescent cells as a novel approach to treating respiratory diseases, and includes repurposing of existing drugs or developing new therapies. Senomorphics, which prevent the development of senescence and inhibit senescence-associated secretory phenotype mediators, include inhibitors of phosphoinositide-3-kinase-mechanistic target of rapamycin signaling, novel antioxidants, and sirtuin activators. Senolytics remove senescent cells by inducing apoptosis and include inhibitors of antiapoptotic proteins, such as B-cell lymphoma-extra large, inhibitors of forkhead box O-4-p53 interaction, heat shock protein 90 inhibitors, and cardiac glycosides. Senotherapies have been effective in animal models of chronic obstructive pulmonary disease and idiopathic pulmonary fibrosis, and several clinical trials are currently underway. The safety of these treatments after long-term administration requires further study, but this could potentially to be a promising approach to treating chronic lung diseases.

**Significance Statement:**

Cellular senescence induced by oxidative stress is a key driving mechanism in chronic lung diseases, such as chronic obstructive pulmonary disease and idiopathic pulmonary fibrosis and may account for disease progression. Senotherapies, including senomorphics that inhibit senescent cells and senolytics that eliminate them, are promising therapeutic approaches to these common diseases, either with repurposed drugs or several new drugs that are in development.

## Introduction

Individuals in every country in the world are living longer, but healthy lifespans (healthspans) are not increasing so that there is a global increase in age-related diseases, resulting in a major impact on health status and quality of life. The World Health Organization estimates that between 2015 and 2050, the proportion of the world’s population over the age of 60 years will almost double from 12% to 22%, resulting in major increasing health and economic burdens (World Health Organization, 2024). Age-related diseases exhibit the classical hallmarks of aging, including genomic instability, telomere shortening, characteristic epigenetic changes, loss of proteostasis, impaired autophagy, deregulated nutrient-sensing, mitochondrial dysfunction, cellular senescence, stem cell exhaustion, altered intercellular communication, chronic inflammation, and dysbiosis (López-Otín et al, 2023). These hallmarks are interconnected and result in acceleration of the normal aging process. Age is a key risk factor for the development of many chronic lung diseases. Accelerated (premature) aging and cellular senescence are now emerging as key mechanism for chronic lung diseases, including chronic obstructive pulmonary disease (COPD), idiopathic pulmonary fibrosis (IPF), cystic fibrosis (CF), bronchiectasis, pulmonary arterial hypertension (PAH), and severe noneosinophilic asthma (Barnes et al, 2019b). These lung diseases are increasing globally and collectively are the third ranked causes of death after cardiovascular disease and cancer (GBD 2019 Chronic Respiratory Diseases Collaborators, 2023). Chronic lung diseases are characterized by acceleration of normal lung aging and the accumulation of senescent cells in the lungs and may drive disease progression. Elderly people may suffer from several age-related diseases and there is increasing evidence that these diseases share common molecular pathways, leading to multimorbidity of the elderly (Yarnall et al, 2017). Multimorbidity results in increased health costs, more hospitalizations, and the risk of drug interactions as a result of polypharmacy (Nicholson et al, 2024). Multimorbidity of the elderly may also account for comorbidities associated with chronic lung diseases, particularly COPD (Barnes, 2015). There is accumulating evidence implicating accelerated lung aging to several chronic lung diseases (Cho and Stout-Delgado, 2020).

The recognition that accelerated aging and cellular senescence are key features of many common age-related diseases has led to a search for new therapeutic approaches called senotherapies. These include drugs that dampen features of cellular senescence (senomorphics or senostatics) and drugs that result in the elimination of senescent cells (senolytics) (Zhang et al, 2023). Senotherapies may be repurposed drugs from other disease indications, such as metformin used to treat type 2 diabetes, or may be newly developed drug entities. Repurposed and novel senotherapies are in clinical trials for a several age-related diseases and may provide a promising new therapeutic approach for several chronic lung diseases (Barnes, 2023). The aim of senotherapies is to extend healthspan and economic predictions estimate that they may have great economic benefits in the future; extension of healthspan by only 1 year is estimated to save a staggering US $40 trillion in global healthcare costs (Scott et al, 2021).

This review focuses on the role of cellular senescence as a driving mechanism for chronic lung diseases, with a particular focus on COPD and IPF and how this may be targeted by existing and novel senotherapies that are in development.

## II Normal lung aging

### A Lung function decline

**Fig. 1:**
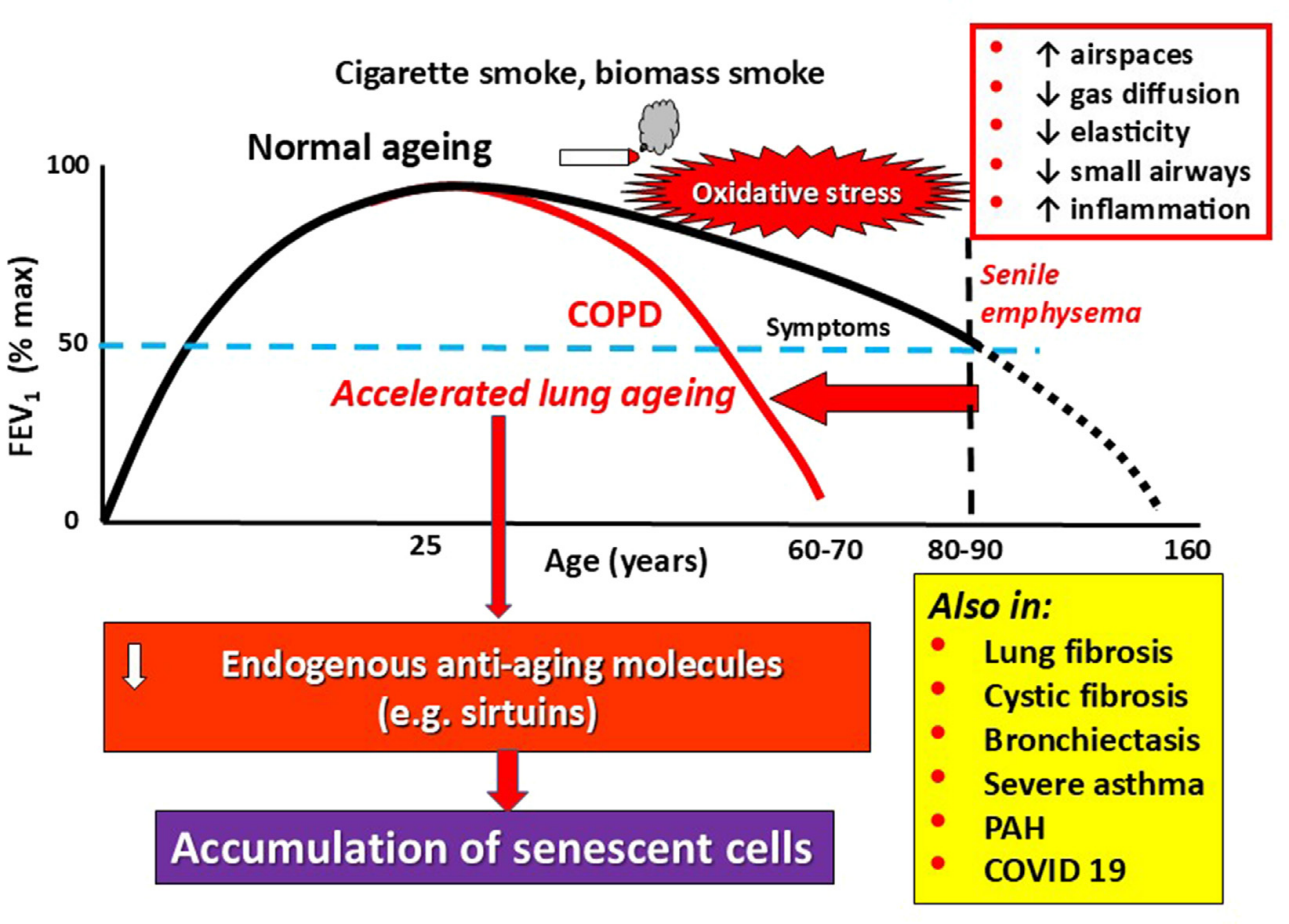
Accelerated lung aging in chronic lung diseases. Lung function reaches a peak at around 25 years and then slowly declines with increasing age, but this does not cause symptoms in normal individuals. The lungs of the very elderly show features of senile emphysema, similar to the pathology of COPD. Patients with COPD have accelerated decline in lung function leading to symptoms when FEV_1_ reaches ∼50% of normal. Accelerated lung aging may be due to loss of endogenous anti-aging molecules, leading to the accumulation of senescent cells in the lung. Cellular senesce is also a feature of other chronic lung diseases.

Lung function usually peaks at around the age of 25 years and then slowly declines with age, but this does not normally lead to functional impairment or respiratory symptoms (Bowdish, 2019). However, in the very elderly there is an increase in alveolar size and a reduction in gas-exchange surface resulting in a loss of elastic recoil and an increase in static lung volumes, sometimes termed “senile emphysema” (Janssens et al, 1999; Cho and Stout-Delgado, 2020). With aging there is a progressive reduction in expiratory flow such as forced expiratory volume in 1 second (FEV_1_), a reduction in the flow volume curve, suggesting small airway obstruction, and increased inhomogeneity of ventilation-perfusion scans, suggesting patchy small airway closure (Fig. 1). The loss of alveolar surface results in a reduction in gas diffusion. There is also a reduction in chest wall compliance and a reduction in dynamic compliance resulting in increased residual volume. However, despite these physiological changes the respiratory system maintains normal oxygenation and normal levels of carbon dioxide in the blood at rest. Respiratory diseases may have a great impact in elderly people due to the reduction in respiratory reserve. Examination of lungs by computed tomography shows an age-dependent loss of small airways, particularly terminal bronchioles, that is correlated with the decline in lung function (Verleden et al, 2021). In addition, there may also be a low-grade inflammation in the peripheral lung (Ascher et al, 2017). There is reduced mucociliary clearance with aging that may increase susceptibility to lung infections and to development of bronchiectasis (Bailey, 2022). Increased susceptibility to lung infections may also reflect reduced immunity associated with aging (immunosenescence) (Yanagi et al, 2017).

### B Immunosenescence

Both innate and adaptive immunity decline with age, and this may contribute to the low-grade inflammation associated with aging (“inflammaging”). Macrophages show changes during aging, with a shift to more inflammatory phenotype (“M1-like”) that may contribute to inflammaging and a defect in phagocytic function (“M2-like”) that may lead to failure to resolve inflammation and increased susceptibility to infections (Moss et al, 2024). The dysregulated innate and adaptive immunity of immunosenescence is an important risk factor for the development of community-acquired pneumonia, with impaired immune responses and bacterial phagocytosis and killing (Boyd and Orihuela, 2011).

Single-cell RNA sequencing has demonstrated that alveolar macrophages from aged mice and humans show senescence phenotypes (Wu et al, 2023). There is also a decrease in neutrophil function with age with defective directed chemotaxis, which has been linked to increased phosphoinositol signaling (Wilson et al, 2020). This neutrophil dysfunction may increase susceptibility to lung infections and is correlated with frailty.

There is an accumulation with aging of long-lived CD28^null^ T-lymphocytes, which are characterized by the loss of the costimulatory molecule CD28. This T-cell subset is characterized by impaired immune responses and is increased in many chronic inflammatory diseases, including COPD and IPF (Habiel et al, 2019; Hodge et al, 2022; Guan et al, 2024). B-lymphocyte function also declines with age and senescent B-cells have impaired antibody production, which may increase susceptibility to infections in the lungs (Frasca, 2018).

## III Cellular senescence

Cellular senescence describes a process in which cells stop dividing and accumulate in tissues (López-Otín et al, 2023). It is a key feature of aging and is closely linked to several other hallmarks of aging, including defective autophagy, mitochondrial dysfunction, and endoplasmic stress, all of which are impaired in chronic lung diseases (Birch et al, 2018; Cloonan et al, 2020; Barnes et al, 2022). Senescent cells accumulate in the lungs with age in humans and mice and this is correlated with deposition of extracellular matrix in aged mice (Calhoun et al, 2016). Cellular senescence has also been described in the lungs of infants with bronchopulmonary dysplasia, which is the most common disease of preterm infants and may be precipitated by hyperoxia from supplementary oxygen delivery (Parikh et al, 2019). Cellular senescence is a state of stable cycle arrest and resistance to apoptosis (Childs et al, 2015; Lucas et al, 2023). Senescent cells are larger and flatter than normal proliferating cells and characteristically stain positively (blue) for senescence-associated *β*-galactosidase (SA-*β*-gal) activity, which is an indicator of lysosomal leak and is active at pH 6. Senescent cells also accumulate insoluble lipofuscin, which is formed from cross-linking of protein aggregates and lipid or sugar residues in the cell (Gorgoulis et al, 2019). Because senescent cells are more resistant to apoptosis they accumulate in tissues over time. These cells are metabolically active and secrete a characteristic array of molecules, many of which are proinflammatory, known as the senescence-associated secretory phenotype (SASP), resulting is a low-grade inflammation. Senescent cells have an increased number of lysosomes and an increased number of fused and abnormal mitochondria linked to impaired autophagy (mitophagy). Stem cells and progenitor cells are particularly susceptible to senescence, resulting in reduced tissue repair and regeneration after injury (López-Otín et al, 2023).

### A Inducers

**Fig. 2:**
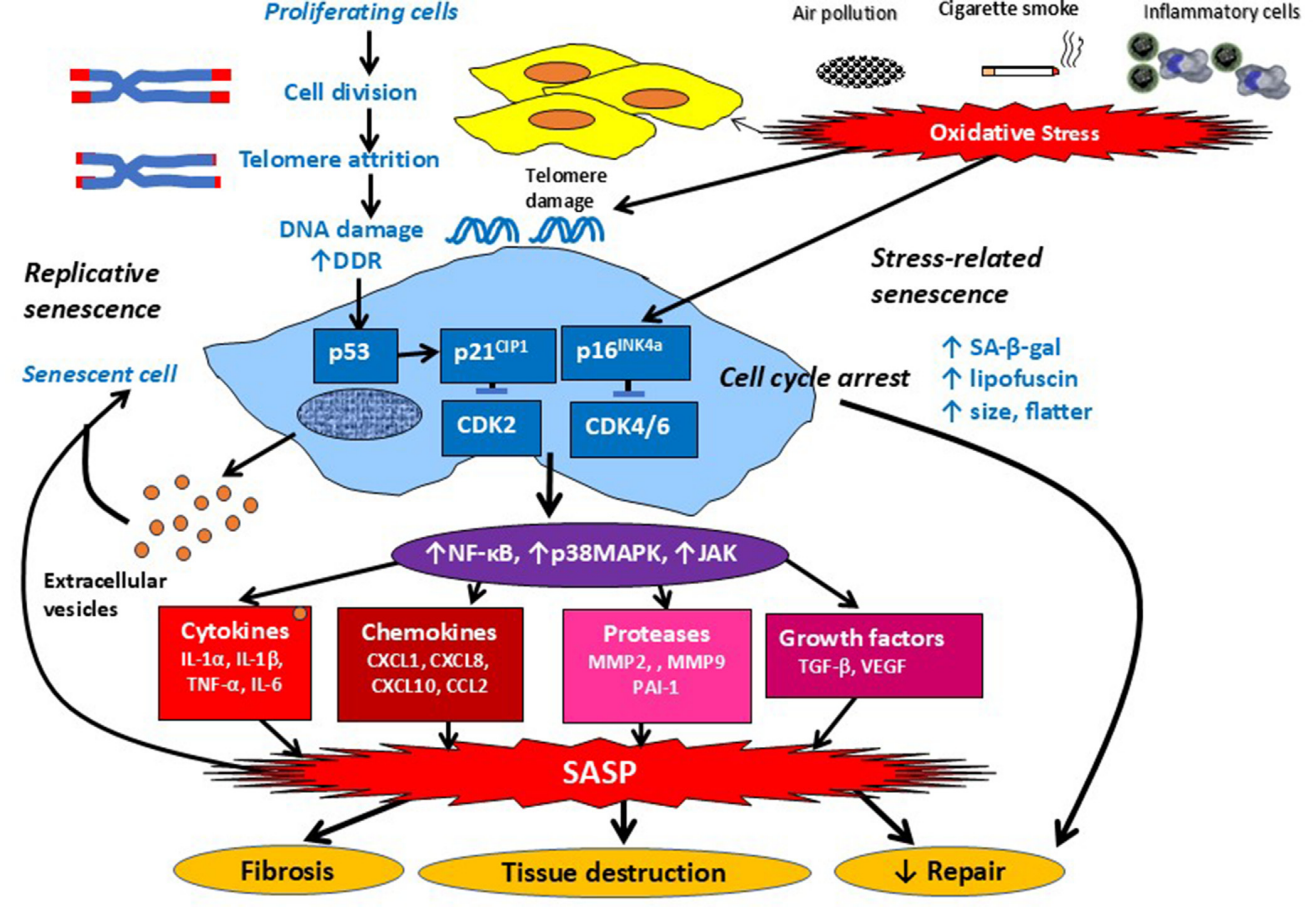
Mechanisms of cellular senescence. Cell division leads to progressive shortening of the telomeres which eventually leads to the activation of the DDR which activates p53, leading to activation of the cyclin kinase inhibitor p21^CIP1^ , which induces cell cycle arrest by inhibiting CDK2 (replicative senescence). Oxidative stress, from cigarette smoke, air pollution, or activated inflammatory cells, may also cause telomere DNA damage and also activates p16^INK4a^ (stress-related senescence), which inhibits CDK4/6 to cause cell cycle arrest. Senescent cells are larger and flatter, and stain positively for SA-*β*-gal and lipofuscin. Senescent cells show activation of NF-*κ*B, p38 MAPK, and JAK, resulting in the secretion of multiple inflammatory proteins known as the SASP, which includes inflammatory cytokines, chemokines, proteins, and growth factors. The SASP induces further senescence. The SASP also leads to structural changes, including local fibrosis and tissue destruction in chronic lung diseases. EVs from senescent cells also induce further senescence, contributing to lung disease progression.

Repeated cellular division results in progressive attrition of telomeres which leads to the activation of the DNA damage response (DDR) pathway, which is characterized by activation of sensor kinases [ataxia-telangiectasia mutated (ATM)/ataxia-telangiectasia Rad3-related] and the formation of DNA damage foci containing phosphorylated histone H2AX and p53 binding protein 1 (53BP1) (Shmulevich and Krizhanovsky, 2021). Prolonged DDR activation stabilizes the tumor suppressor p53, which in turn activates the cyclin-dependent kinase (CDK) inhibitor p21^CIP1^ (also called CDKN1), resulting in cell cycle arrest through inhibition of CDK2/4 and described as replicative senescence (Fig. 2).

Cellular senescence may also be induced by various cell stressors, including oxidative stress (including cigarette smoke and particulate air pollution), ionizing radiation, various oncogenes, and several cytotoxic drugs, including doxorubicin and etoposide (Lucas et al, 2023). These stressors activate a cyclin-dependent kinase inhibitor p16^INK4a^ (CDKN2A) and p15^INK4b^ (CDKN2B), which inhibit CDK4/6 and also in turn activate the tumor suppressor phosphorylated retinoblastoma protein (pRb) pathway, which inhibits cell cycle progression from G1 to S through repression of E2F target genes. These stressors, particularly oxidative stress, may also activate replicative senescence telomeric DNA which is particularly sensitive to oxidative damage (Passos et al, 2007). Both pathways of senescence activate B-cell lymphoma (Bcl)-2 family of antiapoptotic proteins, including Bcl-2, Bcl-X_L_, and Bcl-w, which prevent the cells from entering apoptosis, and therefore removal from tissues by macrophage phagocytosis (efferocytosis) (Basu, 2022).

Cellular senescence plays a role in embryonic development (including lung development) and wound healing through the secretion of growth factors (Demaria et al, 2014). Senescence may also protect against the development of malignancy in younger animals by reducing proliferation of cells that have DNA damage, so that it may promote age-related cancers. However, long-term accumulation of senescent cells during aging results in progressive organ dysfunction, promotion of age-related diseases and malignancy, and a consequent a reduction in lifespan (Lucas et al, 2023). Using a caspase-dependent mechanism to eliminate p16^INK4a^ cells results in a more than 30% extension in lifespan in naturally aging mice due to reduced organ failure and cancer development (Baker et al, 2016a). Transplanting senescent cells into elderly mice leads to the spread of senescence and increased organ failure, frailty, and decreased lifespan, indicating that senescent cells may drive age-related diseases (Xu et al, 2018).

There are several markers that are used in identifying cellular senescence. Senescent cells may be characterized by positive SA-*β*-gal (blue) and lipofuscin staining, increased expression of p21^CIP1^ and p16^INK4a^, and decreased lamin B1, together with markers of DDR, such as ATM kinase, γH2AX, and 53BP1 (Gorgoulis et al, 2019). It is recommended that several of these markers should be used in combination to identify senescent cells.

### B Senescence-associated secretory phenotype

Senescent cells are metabolically active and secrete a wide array of mediators known as the SASP, which result in recruitment of inflammatory cells, perpetuate a chronic low-grade inflammation known as inflammaging, disrupt normal tissue homeostasis, and promote local fibrosis (Birch and Gil, 2020; Ohtani, 2022). Senescent cells are sometimes called “zombie” cells as they persist and release harmful products that contribute to disease progression. SASP mediators may induce further senescence in a paracrine manner (Campisi and d'Adda di Fagagna, 2007). The components of SASP may vary between cells and on the stressor that induces senescence, but is stimulated by p21^CIP1^, which activates p38 mitogen-activated protein kinase (MAPK) and Janus-activated kinases (JAK) and the activation of the proinflammatory transcription factor nuclear factor-*κ*B (NF-*κ*B). This results in the secretion of proinflammatory cytokines [interleukin (IL)-1*β*, IL-6, IL-17A, tumor necrosis factor (TNF)-*α*], chemokines (CXCL1, CXCL8, CCL2), growth factors [vascular-endothelial growth factor, transforming growth factor (TGF-*β*), insulin-like growth factor-1], and matrix metalloproteinases (MMP-2, MMP-9), all of which are increased in age-related diseases. Plasminogen activator inhibitor-1 (PAI-1) is a characteristic SASP mediator and is profibrotic and a biomarker of aging (Vaughan et al, 2017). The SASP also includes activation of the NRLP3 inflammasome, which results in IL-1*β* secretion, further perpetuating the inflammatory response and cellular senescence (Acosta et al, 2013). Single-cell RNA sequencing demonstrates different patterns of gene expression between different cell types after induction of cellular senescence (Wiley et al, 2017). This approach has identified a myriad of distinct cellular states as different cells age, particularly among immune cells (Zhang et al, 2025).

### C Extracellular vesicles

Senescent cells also release extracellular vesicles (EVs) which may contain microRNAs that drive immunosenescence and further cellular senescence (Hanley et al, 2024). Bone-derived EVs from mice change their miRNA composition with age and EVs from aged mice induce stem cells senescence (Davis et al, 2017). Secretion of EVs from senescent cells in the lungs may be an important mechanism driving chronic lung diseases (Kadota et al, 2018). Senescent small airway epithelial cells from patients with COPD release markedly increased numbers of large and EVs, which transmit senescence to normal recipient cells via transfer of microRNA (Devulder et al, 2025). This provides a mechanism for the spread of senescence through the epithelium and to other cell types, such as fibroblasts and AT2 cells resulting in progression of lung diseases, such as COPD. Furthermore, EVs may reach the circulation and taken up by other organs to induce senescence. This may contribute to the development of age-related comorbidities associated with chronic lung diseases, such as COPD.

## IV Senescence in chronic lung disease

### A Chronic obstructive pulmonary disease

**Fig. 3:**
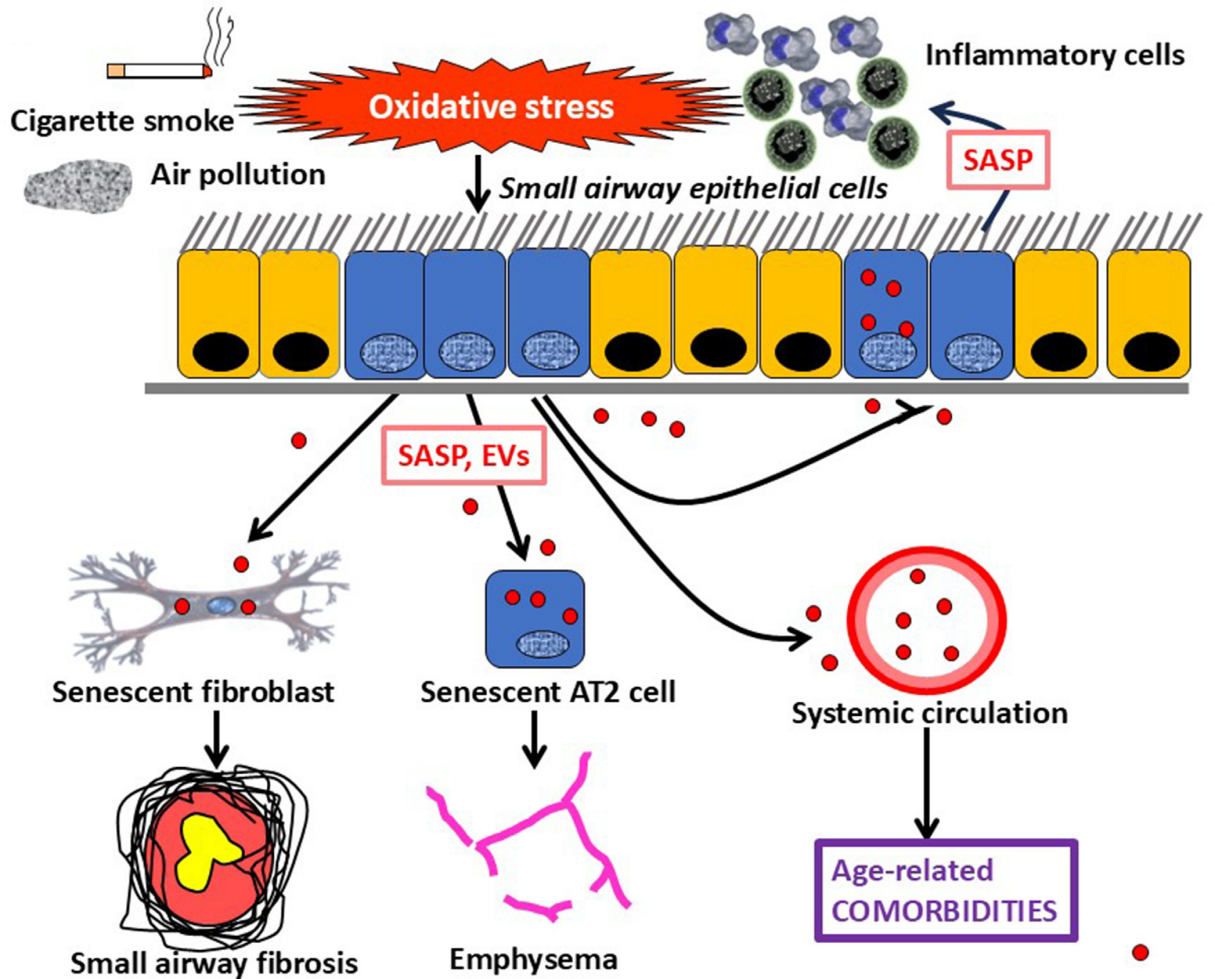
Cellular senescence in COPD. Cigarette smoke, indoor and outdoor air pollution, and chronic inflammation increase oxidative stress to induce senescence of small airway epithelial cells that drive further inflammation because of SASP mediators. Senescent cells also release EVs, which may be taken up by other epithelial cells, by small airway fibroblasts to induce peribronchial fibrosis, and by AT2 cells to result in emphysema, thus resulting in disease progression. In addition, SASP mediators and EVs reach the systemic circulation and may induce senescence in other organs, resulting in the age-related comorbidities commonly seen in patients with COPD.

COPD affects ∼10% of people aged over 45 years and is increasing in prevalence globally as populations age (Barnes et al, 2015; Stolz et al, 2022). Although cigarette smoking is the most common risk factor for COPD, it is also seen in nonsmokers due to exposure to indoor and outdoor air pollution and occupational fumes, particularly in low-middle income countries (Salvi and Barnes, 2009; Sin et al, 2023). COPD is characterized by a low-grade inflammation in peripheral airways and lung parenchyma, which is thought to drive progressive airway obstruction, as well as comorbidities. There is an acceleration of the normal decline in lung function in patients with COPD and increasing evidence that this is due to premature lung aging (Mercado et al, 2015; Barnes, 2017). The inflammation in COPD mirrors that profile of SASP mediators, with increased cytokines (IL-1*β*, IL-6, TNF-*α*), chemokines (CCL2, CXCL1, CXCL8), proteases (MMP-2, MMP-9), and growth factors (TGF-*β*, PAI-1). The characteristic SASP mediator PAI-1 is increased in the sputum, sputum macrophages, and alveoli of patients with COPD and is associated with NF-*κ*B activation (To et al, 2013). This suggests that cellular senescence may be a key driver of chronic inflammation in COPD resulting in recruitment of inflammatory cells, such as monocytes/macrophages and neutrophils into the lung. All cell types in the COPD lung show features of cellular senescence (Tsuji et al, 2006). There is increased senescence of small airway epithelial cells of patients with COPD compared with cells form age-matched controls (Baker et al, 2016b, 2019), as well as in small airway fibroblasts that mediate peribronchiolar fibrosis, which accounts for the early progression of COPD (Wrench et al, 2024) (Fig. 3).

` Defective autophagy may also contribute to cellular senescence in COPD (Barnes et al, 2022) and has been demonstrated in bronchial epithelial cells after exposure to cigarette smoke in vitro and associated with secretion of SASP mediators (Fujii et al, 2012). An important aspect of autophagy is the removal of dysfunctional mitochondria (mitophagy), which accounts for the accumulation of abnormal and fused mitochondria in COPD cells and after exposure to cigarette smoke in vitro (Hoffmann et al, 2013b). There is a reduction in the phosphatase and tensin homolog-deleted from chromosome 10 (PTEN)-induced putative kinase 1-Parkin-E3 ubiquitin protein ligase (PRKN) pathway-mediated which regulates mitochondrial function in COPD epithelial cells and is associated with increased mitochondrial reactive oxygen species (mROS) production (Mizumura et al, 2014; Araya et al, 2019). PRKN knockout mice show enhanced development of emphysema after chronic exposure to cigarette smoke, with accumulation of damaged mitochondria in the lungs and accelerated cellular senescence (Araya et al, 2019). Abnormal mitochondrial function in COPD cells is associated with increased mROS and neutrophilic lung inflammation (Wiegman et al, 2015). Defective autophagy in COPD epithelial cells also results in the accumulation of leaky lysosomes and SA-*β*-gal activation and cellular senescence (Araya et al, 2021). The transcription factor EB (TFEB) is a key regulator of autophagy, mitophagy, and lysophagy (Zhang et al, 2023). Cigarette smoke inhibits TFEB function impairs autophagy in an alveolar macrophage cell line (Xu et al, 2023), whereas induction of TFEB by the peroxisome proliferator activated receptor-α (PPAR*α*) agonist gemfibrozil protect airway epithelia cells from autophagy induced by cigarette smoke exposure in vitro and TFEB knockout mice show impaired autophagy and cellular senescence which can be mitigated by gemfibozil therapy (Bodas et al, 2017).

Oxidative stress is an important inducer of cellular senescence (Finkel and Holbrook, 2000). Cellular senescence in COPD may be activated by chronic oxidative stress in the lungs, which is markedly increased with cigarette smoking and exposure to air pollutants, the major risk factors for COPD (Barnes, 2022). Chronic inflammation in COPD lungs may generate further ROS which further increases oxidative stress to drive more senescence, and this may contribute to disease progression. Increased oxidative stress may result in oxidative damage of DNA in the lungs as evidence by an increase 8-hydroxy-2’-deoxyguanosine and γH2AX in COPD lungs (Caramori et al, 2011). In advanced disease hypoxia, through the activation of the transcription factor hypoxia-induced factor 1*α*, may also induce senescence (Gao et al, 2023). Genetic polymorphisms of telomerase have also been associated with early development of emphysema, although these are usually associated with pulmonary fibrosis (Stanley et al, 2015). Telomere shortening has been described in lung cells form patients with COPD, although these changes are also seen in smokers without airway obstruction (Birch et al, 2015; Ruiz et al, 2021). Both p53/p21 and p16/pRb pathways are activated in COPD lungs and in mice exposed experimentally to cigarette smoke. Deletion of p16 in smoke-exposed mice prevents the development of emphysema (Cottage et al, 2019).

**Fig. 4:**
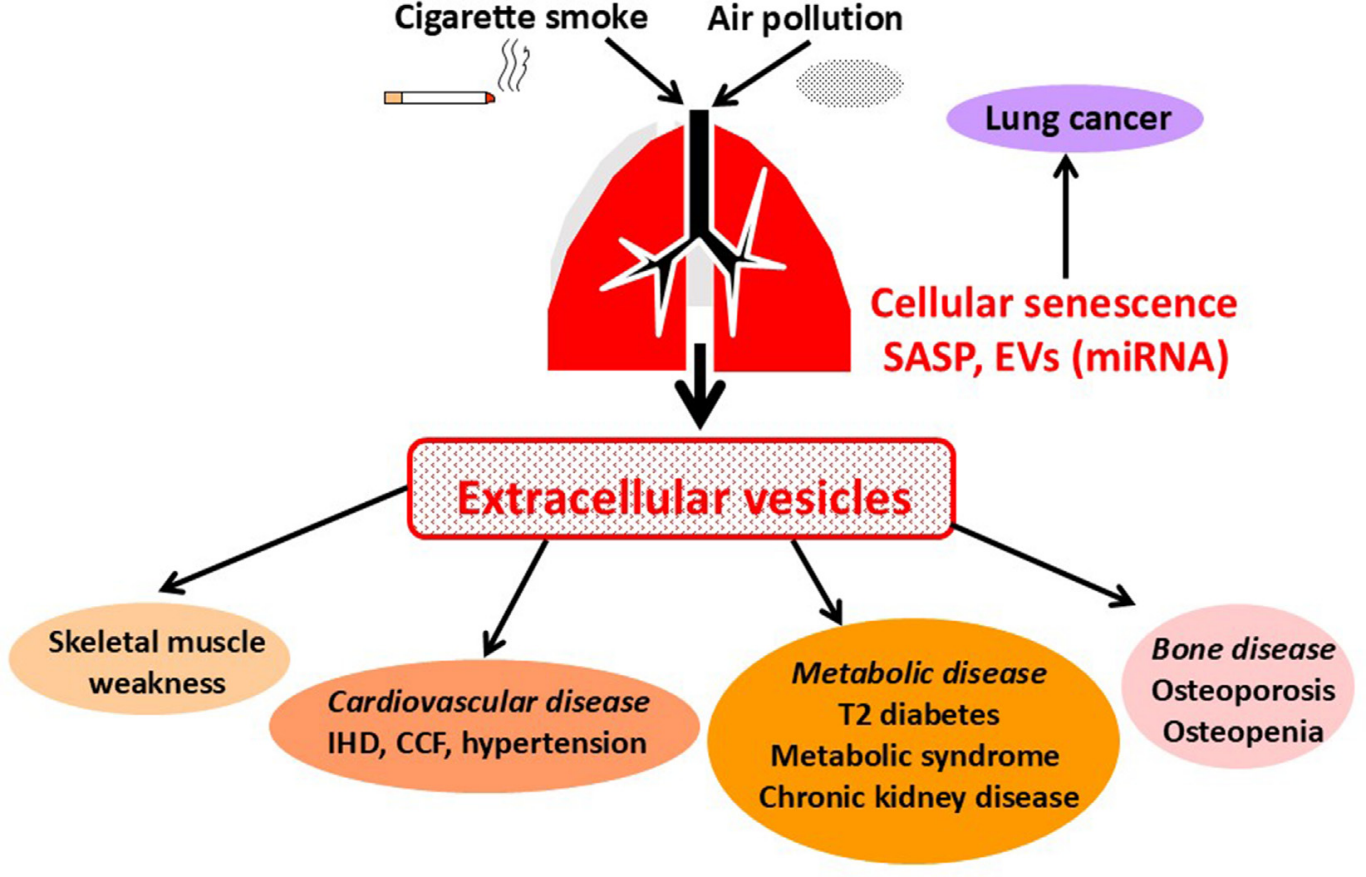
Comorbidities of COPD. Spread of EVs containing microRNAs released from senescent cells in the lung reach the systemic circulation and induce senescence in distant organs, resulting in accelerated age-related skeletal muscle wasting, and cardiovascular, metabolic and bone disease, which are common comorbidities of patients with COPD.

COPD is associated with many comorbidities, particularly cardiovascular and metabolic diseases, that are also diseases of accelerated aging and share common pathways (Fig. 4) (Barnes, 2015; Fabbri et al, 2023). Cellular senescence is also seen in cells outside the lung, such as endothelial progenitor cells that repair vascular damage (Paschalaki et al, 2013). Circulating endothelial progenitor cells from patients with COPD show evidence of DNA damage and biomarkers of senescence and fail to repair vascular injury compared with age-matched control cells; this may account for the high prevalence of cardiovascular disease in patients with COPD.

### B Idiopathic pulmonary fibrosis

IPF is a chronic lung disease with progressive pulmonary fibrosis of unknown cause which usually occurs in elderly individuals and usually leads to death within 2–3 years of diagnosis (Lederer and Martinez, 2018). IPF lungs express many markers of senescence, including p53, p21, and p16, and increased SA-*β*-gal staining and SASP mediators (Alvarez et al, 2017). There is convincing evidence that the pathogenesis of IPF involves senescence of alveolar type 2 (AT2) cells, which lose their progenitor function (Parimon et al, 2023). Lung interstitial fibroblasts also have a senescent phenotype resulting in collagen deposition in the lung (Alvarez et al, 2017). In bleomycin-induced lung fibrosis in mice, elimination of senescent cells restored lung function and physical health but did not reduce fibrosis (Schafer et al, 2017). Single-cell RNA sequencing has identified several senescent cell types in lungs of patients with IPF, including a distinct population of profibrotic alveolar macrophages (Reyfman et al, 2019). IPF is associated with shortened telomeres and gene polymorphisms associated with shorter telomeres account for 85% of genetic variants detected by whole genome sequencing (Armanios, 2012). Telomere shortening or uncapping causes alveolar stem cell dysfunction, and secretin of TGF-*β* as a component of the SASP response, with recruitment and activation of fibroblasts and subsequent fibrosis (Zhang et al, 2021a).

Alveolar senescence is involved in both COPD and IPF but the different responses, with alveolar apoptosis in emphysema and fibrosis in IPF, is poorly understood and suggests that the genetic background and the response to the initiating agents are likely to be important. Some patients have combined pulmonary emphysema and pulmonary fibrosis so it is likely that cellular senescence is a key driving mechanisms in these patients (Alsumrain et al, 2019). Pulmonary fibrosis is also associated with autoimmune diseases, including rheumatoid arthritis. Ionizing radiation to the lung induces cellular senescence and is likely to drive pulmonary fibrosis in these patients and experimentally may be reduced by senolytic therapies (He et al, 2019).

### C Asthma

The role of cellular senescence in asthma is uncertain (Wan et al, 2023). Asthma may start at any age, even in elderly individuals with no previous history. Elderly asthmatic patients may have more severe symptoms and more exacerbations, with a reduced response to corticosteroids. Although asthma is usually associated with type 2 (T2) immunity and increased Th2 and ILC2 cells and eosinophils, asthma in the elderly can be characterized by inflammation driven by non-type 2 (T2) inflammation, involving Th1 and Th17 cells (Nanda et al, 2020). Neutrophilic asthma is likely to be associated with greater oxidative stress in the lungs to promote senescence and other features of aging such as impaired autophagy (Barnes et al, 2022). Mediators that are involved in neutrophilic asthma include IL-6 and CXCL8, which may induce cellular senescence and are themselves prominent components of the SASP (Birch and Gil, 2020). Thymic stromal lymphopoietin is highly expressed in airway epithelial cells of asthmatic patients, particularly in severe disease, and is believed to be a key cytokine driving T2 immunity in asthma. Thymic stromal lymphopoietin induces senescence in airway epithelial cells in vitro, with increased expression of p21 and p16 and increased SA-*β*-gal staining (Wu et al, 2013). Cellular senescence has been described in airway smooth muscle cells and fibroblasts from elderly asthmatic patients, with increased p53, p21, and SASP mediator expression (Aghali et al, 2022). Increased p21 has been described in airway epithelial cells, particularly in patients with severe asthma (Puddicombe et al, 2003). Bronchial fibroblasts from asthmatic patients show evidence of cellular senescence and this is correlated with telomere shortening and suggesting that senescence may play a role in airway remodeling that occurs in severe asthma (Hadj Salem et al, 2015). Shorter telomere length has been described in leukocytes of patients with chronic asthma and correlated with greater eosinophilic inflammation (Belsky et al, 2014).

### D Cystic fibrosis

CF is an autosomal recessive disease due to multiple mutations of the CF transmembrane conductance regulator (CFTR) gene, which impair the function of the CFTR chloride channel (Ong and Ramsey, 2023). CFTR mutations cause disturbance in ion and fluid balance in airways, sweat glands, pancreas, and other organs and result in defective mucociliary function, respiratory infections, and eventually respiratory failure. CF is usually manifest in infancy, affects many organs but particularly the lung, and until recently reduces life expectancy. Advances in antibiotic treatment of lung infections, improved nutrition, and most recently the use of highly effective CFTR modulator drug combinations (particularly ivacaftor/tezacaftor/electocaftor for the common F508del variant) has markedly extended the median lifespan on patients with CF from 36 years in 2006 to 53 years in 2021, with a marked improvement in lung function and decreased exacerbations. With early treatment with CFTR modulators it is predicted that patients with CF may now be expected to reach the median age of 83 years (Lopez et al, 2023). This means that lung aging may have an important influence in CF. The chronic neutrophilic inflammation in patients with CF generates high levels of oxidative stress in the lungs which may drive senescence. Primary bronchial epithelial cells from patients with CF show increased DNA damage markers (γH2AX) and increased p16, which may be induced by neutrophil elastase in normal bronchial epithelial cells (Fischer et al, 2013). The intense neutrophilic inflammation in CF airways may be a potent inducer of cellular senescence and telomere dysfunction through increased local oxidative stress (Lagnado et al, 2021). Mitochondrial function is also defective in CF epithelial cells and may further increase oxidative stress in the airways (Antigny et al, 2009). SASP mediators, including IL-6, CXCL1, and CXCL8, are also prominent in CF airways (Bezzerri et al, 2019).

### E Bronchiectasis

Non-CF bronchiectasis is a chronic lung disease characterized by cough, purulent sputum production, and exacerbations, which is associated with irreversible dilatation of bronchi (Chalmers et al, 2023). Airway epithelial cells in patients with bronchiectasis show increased p21 expression, increased γH2AX indicating activation of the DDR, and increased telomere associated DNA damage (Birch et al, 2016). Telomere length is also reduced in sputum cells of patients with bronchiectasis (Lim et al, 2022). This suggests that cellular senescence is a feature of bronchiectasis and may contribute to the chronic inflammation.

### F Pulmonary arterial hypertension

PAH is a rare disease of the pulmonary vasculature with increased pulmonary arterial pressure and characterized by pulmonary vascular smooth muscle proliferation and endothelial dysfunction (Mocumbi et al, 2024). There is increasing evidence that cellular senescence may be contributory to the development of type 1 (idiopathic) PAH, with increased expression of p21, p16, and the DNA damage markers γH2AX and 53BP1 in pulmonary endothelial and vascular smooth muscle cells (Kyi et al, 2022; Born et al, 2023). Mice exposed to hypoxia develop pulmonary hypertension and increased senescent cells expressing p16 and DNA damage markers, with increased release of SASP mediators IL-6, CCL2, CXCL8, and PAI-1 (Born et al, 2023). In patients with COPD with secondary PAH, there is an increase in SA-*β*-gal staining, p21 and p16 expression in pulmonary vascular smooth muscle cells, compared with patients with COPD without PAH (Noureddine et al, 2011). Furthermore, telomere shortening is correlated with the severity of PAH in patients with COPD. However, elimination of senescent cells in murine models of PAH in mice paradoxically worsens vascular remodeling and pulmonary hemodynamics, suggesting that senescent cells in these models may even be protective (Born et al, 2023).

### G COVID-19

COVID-19 is a respiratory infection caused by SARS-CoV-2 that may lead to acute respiratory distress syndrome and systemic inflammation. COVID-19 has a greater impact in elderly people and there is increasing evidence that cellular senescence may be involved in the inflammatory response (Schmitt et al, 2023). In patients who died from acute respiratory distress syndrome as a result of SARS-CoV-2 infection the virus predominantly affects AT2 cells, which show features of cellular senescence and secrete SASP mediators (Evangelou et al, 2022; Havaki et al, 2022). SARS-CoV-2 induces cellular senescence and the release of SASP mediators, such as IL-1*β* and IL-6, similar to the increased cytokines (“cytokine storm”) seen in severe COVID-19, in a human epithelial cell line (Evangelou et al, 2022). A hamster model of COVID-19 shows cellular senescence in the lungs when infected with SARS-CoV-2 and the cytokine storm and systemic inflammation induced is abrogated by senolytic therapies (Lee et al, 2021b). Cellular senescence may also be a mechanism accounting for some post-COVID sequalae (“long COVID”) because persistence of senescent cells in the lung or at extrapulmonary sites may account for sustained increases in cytokines (Tsuji et al, 2022; Liew et al, 2024). Several clinical trials with a senolytic therapy (fisetin) in severe COVID-19 are underway (Verdoorn et al, 2021).

Cellular senescence may play an important role in the pathogenesis of other viral pneumonias, such as influenza (Torrance et al, 2024). Immunosenescence may explain why vaccines against viral infections, such as COVID-19 and influenza, are less effective in the elderly. This suggests that senotherapies may be useful for enhancing vaccine efficacy in elderly individuals and improving immune function (Bracken et al, 2025).

## V Antiaging molecules

Cellular senescence is counteracted by several antiaging molecules and there is increasing evidence that impairment of these regulatory molecules may accelerate the aging process in lungs, contributing to the persistence of chronic lung diseases (Mercado et al, 2015).

### A Sirtuins

Silent information regulators or sirtuins are NAD^+^-dependent protein deacetylases that were discovered in yeast but are highly conserved across all species. Seven mammalian homologues (SIRT1-7) of yeast Sir2 have been identified and play a key regulatory role in cell metabolism, inflammation, oxidative stress, cell proliferation, and senescence and apoptosis (Wu et al, 2022). In particular, SIRT1 and SIRT6 have been linked to extension of lifespan in several species (You and Liang, 2023). Among the other sirtuins, SIRT3, SIRT4, and SIRT5 are localized to mitochondria and defects in their activity are associated with mitochondrial dysfunction and cellular senescence (Ji et al, 2022).

#### 1 SIRT1

SIRT1 deacetylates several transcription factors and regulatory proteins that are involved in inflammation, antioxidant expression, DNA repair, mitochondrial function, proteostasis, and autophagy. SIRT1 inhibits p53-induced senescence, activates the transcription factor forkhead box O (FOXO)3a, which regulates antioxidant genes (superoxide dismutases and catalase), activates PPARγ coactivator-1α, a transcription factor that regulates and maintains mitochondrial function, and inhibits activated (acetylated) NF-*κ*B, thereby suppressing SASP mediator secretion. SIRT1 inhibits cellular senescence and restores defective autophagy associated with cellular senescence. SIRT1 suppresses lung adenocarcinomas driven by K-Ras, indicating that reduced SIRT1 may also increase the risk of lung cancer (Costa-Machado et al, 2018).

#### 2 SIRT6

SIRT6 is an ADP-ribosylase as well as a protein deacetylase and has also been implicated in age-related diseases (You and Liang, 2023). SIRT6 regulates telomere length, DNA repair, and NF-*κ*B, metabolic homeostasis, and extends lifespan (Kugel and Mostoslavsky, 2014). SIRT6 also activates the transcription factor Nrf2, which is an important regulator of multiple antioxidant genes (Pan et al, 2016). SIRT6 knockout mice show premature aging and genomic instability, whereas overexpression of SIRT6 prolongs lifespan (Tian et al, 2019). SIRT6 interacts with lamin A/C, mutations of which are associated with certain human progeria syndromes. A genetic variant of SIRT6 with increased activity and interaction with lamin A/C has been described in centenarians (Simon et al, 2022).

#### 3 Reduced sirtuins in chronic obstructive pulmonary disease

SIRT1 mRNA and protein are reduced in peripheral lung, airway epithelial cells, and circulating peripheral blood mononuclear cells of patients with COPD (Rajendrasozhan et al, 2008; Nakamaru et al, 2009; Baker et al, 2016b). Serum SIRT1 is also reduced in patients with COPD (Yanagisawa et al, 2017b). SIRT1 inhibits the mechanistic target of rapamycin (mTOR) and thereby increases autophagy, so a reduction in SIRT1 activity contributes to impaired autophagy in COPD. Oxidative stress in COPD reduces activity and expression of PTEN (Yanagisawa et al, 2017a), which activates the phosphoinositide-3-kinase (PI3K)-mTOR pathway, resulting in a reduction in SIRT1. SIRT6 mRNA and protein expression are also reduced in COPD lungs and small airway epithelial cells (Nakamaru et al, 2009; Baker et al, 2016b). In airway epithelial cells SIRT6 is reduced by cigarette smoke exposure, leading to senescence and impaired autophagy (Takasaka et al, 2014). Skeletal muscle wasting is common in more severe patients with COPD and muscle cells show evidence of senescence with increased p21 and p16, with a reduction in SIRT1/6 (Lakhdar et al, 2018). T-lymphocyte senescence (CD^28null^CD8^+^) in patients with COPD is also associated with a reduction in SIRT1 (Hodge et al, 2020). In IPF an increase in SIRT6 expression in airway epithelial cells, which may be induced in vitro by TGF-*β*, although overexpression of SIRT6 inhibits cellular senescence in these cells via proteasomal degradation of p21 and myofibroblast differentiation to fibroblasts (Minagawa et al, 2011).

**Fig. 5:**
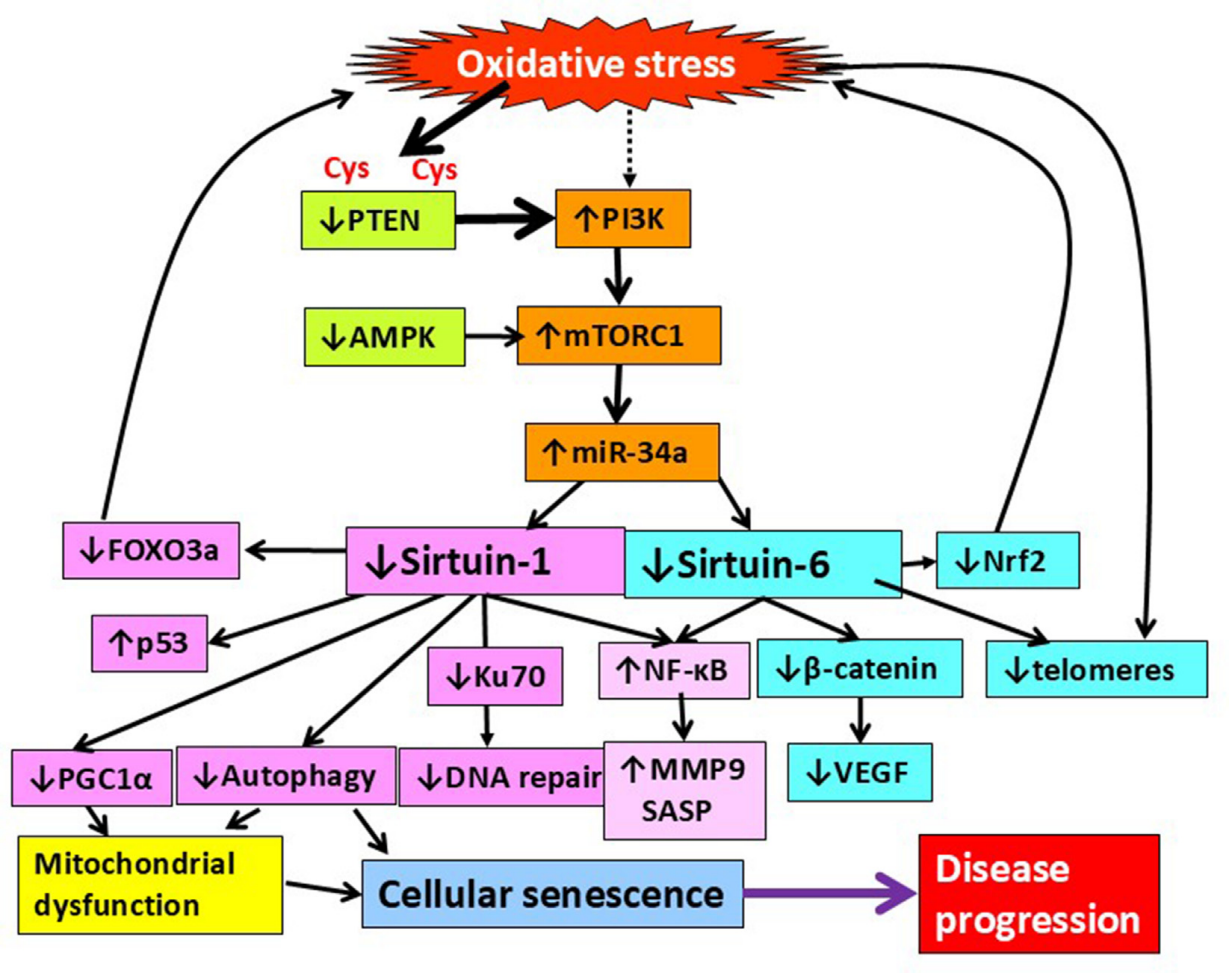
Reduced sirtuins in chronic lung disease. Oxidative stress inactivates PTEN, which has cysteine (Cys) residues at its catalytic site, resulting in the activation of PI3K and mTORC1. This activates microRNA-34a, which inhibits sirtuin-1 and sirtuin-6 in parallel. Reduced sirtuin-1 leads to cellular senescence and mitochondrial dysfunction. Reduction of sirtuin-1 and sirtuin-6 leads to secretion of the SASP, which spreads senescence leading to disease progression. Reduction in sirtuin-1 and sirtuin 6 leads to reduced antioxidant genes FOXO3a and Nrf2, which further increases oxidative stress to accelerate the aging process.

SIRT1 and SIRT6 (but not other sirtuins) are regulated by miR-34a, which binds to the 3’ untranslated regulatory regions of SIRT1/6 mRNA, reducing mRNA and protein levels. MiR-34a is increased in peripheral lungs and epithelial cells of patients with COPD, and this is correlated with increased expression of senescence markers in lung cells (Baker et al, 2016b). MiR-34a is increased by oxidative stress through activation of PI3K-mTOR signaling resulting in a parallel reduction in SIRT1 and SIRT6 (Fig. 5), whereas other sirtuins are unchanged, as in COPD lungs. An antagomir of miR-34a, which blocks its action, restores SIRT1 and SIRT6 in senescent small airway epithelial cells from patients with COPD, reduces markers of cellular senescence (p16, p21, and p53), reduces the SASP response (TNF-*α*, IL-1*β*, IL-6, CCL2, CXCL8, and MMP9), and increases proliferation of senescent epithelial cells by reversing cell cycle arrest (Baker et al, 2016b). MiR-34a is also increased in COPD macrophages and may be associated with impaired phagocytosis and uptake of apoptotic cells (efferocytosis) observed in this disease (McCubbrey et al, 2016). Another miRNA, miR-570, also inhibits SIRT1 (but not SIRT6) and is activated via p38 MAP kinase and AP-1. MiR-570 is increased in COPD peripheral lung and small airway epithelial cells. An antagomir restores sirtuin-1, reduces senescence markers, and reverses cell cycle arrest (Baker et al, 2019).

#### 4 Reduced sirtuins in other lung diseases

Sirtuins have been documented as key regulators of pulmonary fibrosis (Mazumder et al, 2020). Cigarette smoke, a well documented risk factor for IPF, reduces SIRT1 and induces senescence in AT2 cells (Zhang et al, 2021c). A decrease in SIRT1 has been described in serum and blood leukocytes of patients with IPF. IPF is also associated with a reduction in SIRT3, which is linked to AT2 mitochondrial dysfunction and the promotion of fibrosis (Jablonski et al, 2017). Defective sirtuin function has also been associated with asthma (Liu and Shi, 2022). A reduction in SIRT1 is reported in bronchoalveolar lavage fluid of ovalbumin-sensitized and exposed mice and is associated with T2 inflammation (Wang et al, 2015). Reduced SIRT1 in T cells activates GATA3, which increases expression of T2 cytokines and promotes eosinophilic inflammation (Colley et al, 2016). A defect in SIRT1 has also been implicated in virally induced asthma exacerbations (Fukuda et al, 2020). SIRT1 is also reduced in airway epithelial cells and peripheral blood mononuclear cells of patients with bronchiectasis, and this is independent of disease severity (Han et al, 2021). SIRT1 is reduced by growth factors in human pulmonary artery smooth muscle cells in vitro and in a monocrotaline-induced pulmonary hypertension in rats, whereas SIRT1 inhibits smooth muscle proliferation (Zhou et al, 2015).

### B Klotho

Klotho is a transmembrane *β*-glucuronidase which acts as a coreceptor for fibroblast growth factor-23 and is protective against aging (Prud'homme et al, 2022). Mice deficient in the Klotho gene show accelerated aging and develop spontaneous emphysema (Suga et al, 2000). Even Klotho heterozygotes develop emphysema later in life. Klotho mice are also more susceptible to developing emphysema after cigarette smoke (Sato et al, 2007). Klotho expression is reduced by supernatants from senescent cells and mediated in part by secretion of activin and IL-1*α* (Zhu et al, 2022).

Serum Klotho concentrations are reduced in patients with COPD (Shi et al, 2024). Klotho expression is reduced in airway epithelial cells of patients with COPD and is reduced in airway epithelial cells by cigarette smoke extract and TNF-*α* in vitro; Klotho knockdown also increases the secretion of inflammatory mediators and oxidative stress (Gao et al, 2015). Klotho is also reduced in peripheral blood monocytes and alveolar macrophages of patients with COPD, and knockdown of endogenous Klotho increases secretion of inflammatory mediators, such as IL-6, TNF-*α*, and MMP9 (Li et al, 2015).

Klotho is reduced in plasma and pulmonary fibroblasts of patients with IPF and in mice exposed to bleomycin. Conversely, transgenic mice overexpressing Klotho are protected from the development of pulmonary fibrosis after bleomycin (Barnes et al, 2019a). Analysis of a large IPF RNA-seq database has identified Klotho as a key regulator and recombinant Klotho is effective in preventing lung fibrosis in the bleomycin mouse model (Huang et al, 2020). In the monocrotaline induced pulmonary hypertension model in rats, whereas Klotho abrogated the development of pulmonary hypertension in these animals (Varshney et al, 2016).

### C Other antiaging molecules

Many other endogenous molecules have antiaging effects, including HDAC2, Nrf2, and FOXO-3a, all of which are reduced in COPD lungs. FOXO-3a and Nrf2 regulate multiple antioxidant genes in the lungs and regulated by SIRT1 and SIRT6 respectively (Motta et al, 2004; Liu et al, 2023a). Senescence marker protein-30 is involved in calcium regulation and is reduced in the lungs of patients with COPD. Senescence marker protein-30 knockout in mice leads to alveolar enlargement and accelerated emphysema due to cigarette smoke (Sato et al, 2006).

## VI Senomorphic drugs

**Fig. 6:**
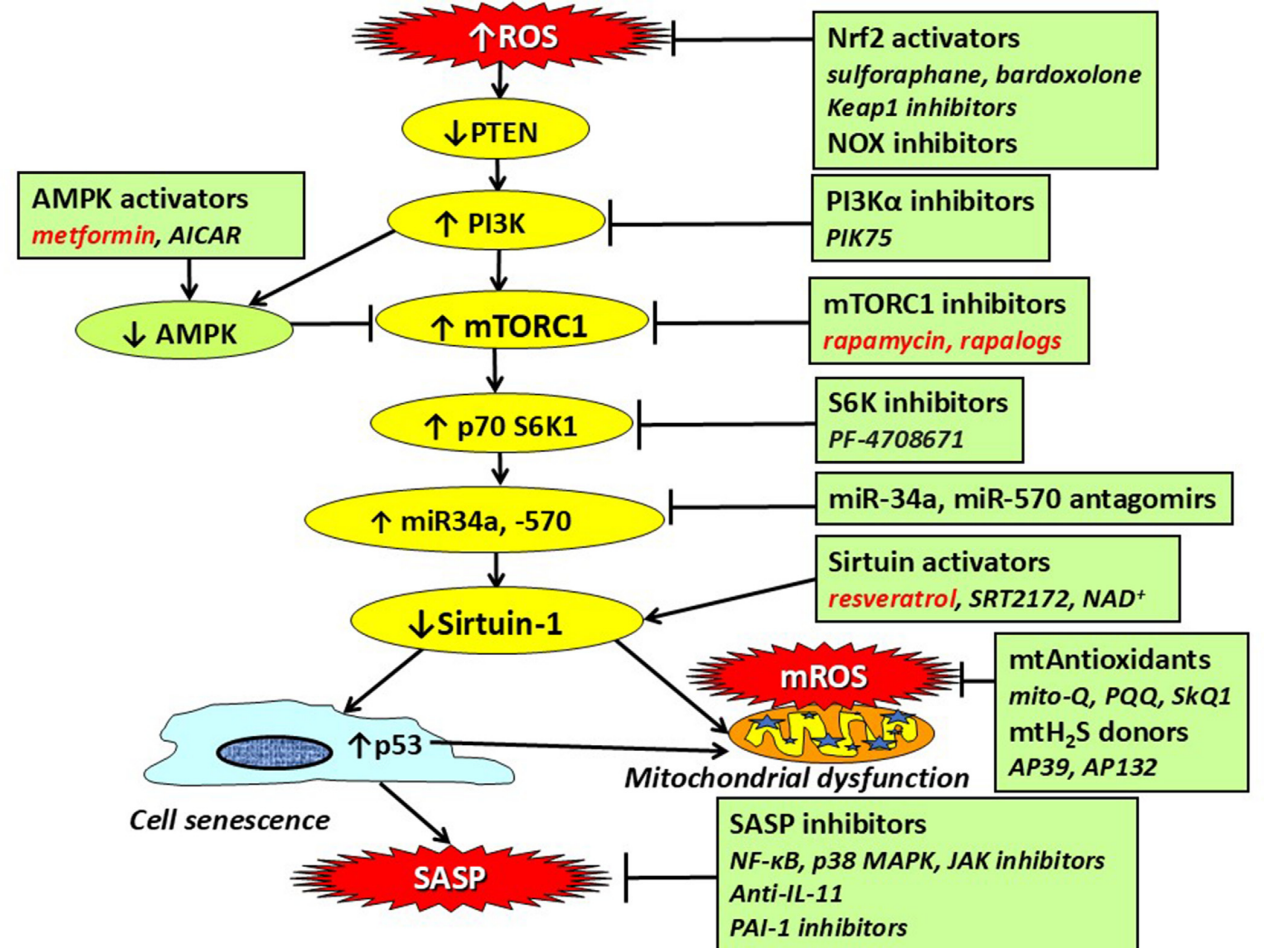
Senomorphic drugs for chronic lung disease. ROS inhibit PTEN, resulting in activation of PI3K and then mTORC1, which is inhibited by AMPK. mTOR activation reduces sirtuin-1, resulting in cellular senescence and mitochondrial dysfunction, which lead to release of mROS. Senescent cells release inflammatory proteins known the SASP. These pathways can be inhibited at several points as shown in the boxes. The drugs in red have already been tested in clinical studies.

Senomorphic drugs act by inhibiting the harmful effects of cellular senescence, such as inhibiting the SASP response (Zhang et al, 2023). Several classes of drug have been described as senomorphics, but the most widely studied are inhibitors of the PI3K-mTOR signaling pathway which promotes cellular senescence, inhibits autophagy, and reduces SIRT1/6 (Fig. 6; Table 1).

**Table 1:** Senomorphic drugs that have been studied in lung disease in vitro and in vivo

Target	Drug	In Vitro	In Vivo
mTORC1	Rapamycin	+	+
(Korfhagen et al, 2009; Jin et al, 2014; Houssaini et al, 2018; Gomez-Manjarres et al, 2023)			
	Everolimus	+	+
(Malouf et al, 2011; Mannick et al, 2014)			
mTORC1/PI3K	Omipalisib	+	+
(Mercer et al, 2016; Lukey et al, 2019)			
AMPK	Metformin	+	+
(Cheng et al, 2017; Rangarajan et al, 2018; Spagnolo et al, 2018; Kheirollahi et al, 2019; Saito et al, 2019; Polverino et al, 2021)			
IL-11	Anti-IL-11	+	+
(Ma et al, 2022; Widjaja et al, 2024)			
NF-*κ*B	SR12343	+	+
(Zhang et al, 2021b)			
p38 MAPK	SB203580	+	
(Freund et al, 2011; Alimbetov et al, 2016)			
JAK	Ruxolitinib	+	+
(Xu et al, 2015; Griveau et al, 2020)			
	Fedralinib	+	+
(Ruan et al, 2021)			
ATM kinase	KU55933	+	+
(Paschalaki et al, 2013; Zhao et al, 2020)			
Sirtuin activation	Resveratrol	+	+
(Culpitt et al, 2003; Birrell et al, 2005; Paschalaki et al, 2013; Navarro et al, 2017; Wang et al, 2017; Beijers et al, 2020)			
STAC		+	+
(Nakamaru et al, 2009; Yao et al, 2014; Zeng et al, 2017; Gu et al, 2020)			
miR	miR-34a antagomir	+	
(Baker et al, 2016b)			
	miR-570 antagomir	+	
(Baker et al, 2019)			
Nrf-2	Bardoxolone	+	+
(Sussan et al, 2009)			
	Dimethyl fumarate	+	
(Cattani-Cavalieri et al, 2020)			
Nox	Apocynin	+	+
(Stefanska et al, 2012; Oostwoud et al, 2016)			
Nox4	Setanaxib	+	+
(Ghatak et al, 2017; Huang et al, 2017)			
mROS	miti-Q	+	+
(Hara et al, 2013; Wiegman et al, 2015; Belchamber et al, 2019)			
H_2_S	AP39	+	+
(Pacitti et al, 2021)			

### A Inhibiting phosphoinositide-3-kinase-mechanistic target of rapamycin signaling

PI3K-mTOR signaling can be activated by oxidative stress. Oxidative stress inactivates PTEN by oxidizing cysteine residues at its catalytic site, leading to reduced expression and activity in COPD lungs and epithelial cells. This results in the activation of PI3K-mTOR signaling (Yanagisawa et al, 2017a). Although activators of PTEN have been developed for treating malignancy, none have yet been clinically approved (Boosani et al, 2019). Several PI3K inhibitors have been developed, including nonselective and isoform selective drugs. The isoform of PI3K that is activated in senescence is PI3K*α*, but so far inhibitors of PI3K*α*, which were developed for treating cancer, have been ineffective and with major side effects (Elmenier et al, 2019; Hanker et al, 2019). A PI3K inhibitor targeted to a fibroblast activation protein that is only expressed at sites of fibrosis inhibited collagen production from IPF fibroblasts and inhibited bleomycin-induced pulmonary fibrosis in mice (Hettiarachchi et al, 2020). PI3K activation stimulates the mTOR complex (mTORC1), which shows activity in COPD lung and epithelial cells and may be a key driver of senescence (Mitani et al, 2016; Houssaini et al, 2018). Several mTOR inhibitors have been developed and some are already in clinical use as immunosuppressants.

#### 1 Rapamycin and rapalogs

Rapamycin (sirolimus) is a macrolide that was isolated from a species of *Streptomyces* bacteria discovered in Easter Island, and currently used as an immunosuppressant and in the treatment of lymphangioleiomyomatosis and transplantation. It appears to ameliorate several age-related disease models in mice and extend lifespan in several species (Selvarani et al, 2021). Rapamycin inhibits mTOR, a conserved serine-threonine kinase, that is an important regulator of cellular metabolism, senescence, and autophagy. Low-dose rapamycin reduces SA-*β*-gal positive cells and secretion of SASP mediators in mice exposed to cigarette smoke (Houssaini et al, 2018). mTOR and its downstream target tuberose sclerosis 2 show increased expression in airway epithelial cells from patients with COPD and are induced by cigarette smoke Unfortunately, rapamycin has several adverse effects related to immunosuppression and abnormal lipid metabolism, some of which are related to its inhibition of mTORC2 and this has led to the development of less toxic analogues called rapalogs, such as everolimus and temsirolimus. Low-dose everolimus reduces immunosenescence in elderly people and may reduce respiratory infections (Mannick et al, 2014, 2018). Because low doses are effective and relatively well tolerated in elderly people, this might be a suitable long-term treatment for COPD and further extended studies are warranted. More selective mTORC1 inhibitors are also in development, which appear to have less adverse effects, such as lipid dysregulation and so may be better tolerated (Schreiber et al, 2019).

Rapamycin has contradictory effects in animal models of pulmonary fibrosis. Rapamycin attenuates bleomycin-induced pulmonary fibrosis in rats, with reduced expression of MMP9 (Jin et al, 2014), whereas in mice it appears to be ineffective (Madala et al, 2011). However, rapamycin inhibits the development of pulmonary fibrosis in a mouse model overexpressing TGF-*β* (Korfhagen et al, 2009) and protects against the development of pulmonary fibrosis after paraquat exposure in mice (Xu et al, 2017). A clinical trial of everolimus in patients with IPF showed clinical deterioration (Malouf et al, 2011). In an exploratory trial in patients with IPF, omipalisib (GSK2126458), a potent inhibitor of PI3K/mTOR, inhibited PI3K and reduced uptake of ^18^F-2*-*fluoro-2-deoxy-d-glucose positron emission tomography/computed tomography scans in fibrotic areas of lungs (Lukey et al, 2019). The same drug inhibits IPF lung-derived fibroblasts in vitro (Mercer et al, 2016). In patients with IPF, rapamycin reduces circulating fibrocytes, which are believed to home to areas of fibrosis in the lungs (Gomez-Manjarres et al, 2023).

#### 2 Metformin and AMP kinase activators

AMP kinase (AMPK) is an endogenous inhibitor of mTORC1 and is reduced in COPD (Cheng et al, 2017). AMPK activators reduce cellular senescence and SASP in animal models of chronic inflammatory diseases and also extend lifespan (Martin-Montalvo et al, 2013; Kulkarni et al, 2020). The AMP activator adenosine analogue 5-aminoimidazole-4-carboxamide riboside reduces cellular senescence induced by cigarette smoke extract in human small airway epithelial cells in vitro. Knockdown of AMPK*α* increases senescence markers (p16, p21) and SASP mediators in BEAS-2B epithelial cells (Cheng et al, 2017).

Metformin, a biguanide widely used to treat type 2 diabetes, also activates AMPK (among other actions), reduces cellular senescence, and extends the lifespan of several species, including mammals (Kulkarni et al, 2020). Metformin reduces the SASP response in airway epithelial cells in vitro and abrogates the development of elastase-induced emphysema in mice (Cheng et al, 2017; Saito et al, 2019). A large prospective study, TAME (Targeting Aging with Metformin), is currently underway to investigate the effects of metformin on hallmarks of aging and in several chronic diseases (Kulkarni et al, 2020). Metformin protects against the development of emphysema in mice exposed to cigarette smoke over 6 months, decreased neutrophilic inflammation, and decreased γH2AX and markers of endoplasmic reticulim stress, with an increase in SIRT1 and increased phosphorylation (activation) of AMPK. Metformin reduces mTOR activation by cigarette smoke in human bronchial epithelial cells (Polverino et al, 2021). Metformin is relatively well tolerated in patients with COPD, although it may cause gastrointestinal side effects and an increase in plasma lactate concentration (Hitchings et al, 2015, 2016). No long-term clinical trials of metformin in COPD have been reported, but its use appears to reduce mortality in patients with COPD compared with matched patients not using metformin (Yen et al, 2018). A meta-analysis showed that patients with COPD treated with metformin for type 2 diabetes showed reduced exacerbations and mortality compared with age-matched controls (Zhu et al, 2019). In a longitudinal follow-up study of patients with COPD, there was a reduced decline in diffusion capacity but not in FEV_1_ decline in patients taking metformin (Kahnert et al, 2022). In the COPDGene cohort of patients with COPD, treatment with metformin reduced progression of emphysema measured by computed tomography scan (Polverino et al, 2021). More long-term controlled studies of metformin in COPD are needed.

AMPK activity is reduced in lungs of mice with bleomycin-induced pulmonary fibrosis and in fibrotic areas of lungs from patients with IPF. Metformin activates AMPK in myofibroblasts of IPF patients and reduces fibrosis and is also effective in accelerating the resolution of lung fibrosis in the bleomycin model in mice (Rangarajan et al, 2018). Metformin appears to reverse fibrosis by inhibiting the effects of TGF-*β* on collagen formation through promoting the transformation of myofibroblasts to lipofibroblasts (Kheirollahi et al, 2019). However, a retrospective analysis showed that patients with IPF treated with metformin for diabetes had no difference in clinical outcomes compared with untreated patients, although the number of patients (*n* = 71, 11%) was relatively small (Spagnolo et al, 2018). In a large database study, patients with IPF treated with metformin for diabetes showed a reduced mortality and reduced hospitalizations compared with patients not treated with this drug (Teague et al, 2022). In a murine model of allergic asthma metformin reduces inflammation and airway remodeling in response to inhaled allergen and correlated with activation of AMPK*α* in the lungs (Ma et al, 2022). Metformin also reduces exacerbations in patients with coexistent asthma and COPD (Wu et al, 2021). In patients with asthma and type 2 diabetes, metformin reduces exacerbations and hospitalizations (Li et al, 2016). Overall, metformin appears to be promising as a senomorphic drug, as it is relatively well tolerated, although it may have other mechanisms of action in addition to its activation of AMPK. More controlled clinical trials in chronic lung diseases are certainly indicated.

Because metformin is not specific for activation of AMPK there has been a search for more selective activators with fewer off-target effects and several novel activators are now in development (Steinberg and Carling, 2019). Lixumistat (IM156) is a biguanide related in structure to metformin but with greater potency. Lixumistat reverses pulmonary fibrosis in the bleomycin model and inhibits human lung fibroblasts (Willette et al, 2021). This drug is now in clinical trials for cancer but no studies in chronic lung disease have been reported.

#### 3 S6 kinase inhibitors

Downstream of mTOR is p70 ribosomal protein S6 kinase-1 (S6K1), which promotes cellular senescence and reduces SIRT1 (Fumagalli and Pende, 2022). Deletion of S6K1 prolongs the lifespan of mice (Selman et al, 2009) and reduces liver inflammation, but does not appear to reduce senescence (Gallage et al, 2024). S6K1 is activated in COPD lungs (Mitani et al, 2016). Several S6K1 inhibitors are in preclinical and clinical development for treatment of cancers but have not yet been studies in chronic lung disease (Artemenko et al, 2022). PF-4708671, a highly selective S6K1 inhibitor, inhibits irreversible cell cycle arrest in a cell line (Leontieva et al, 2013).

### B Senescence-associated secretory phenotype inhibitors

#### 1 Mediator inhibitors

As SASP mediators appear to amplify and spread cellular senescence inhibiting their production and effects may have senomorphic effects. CXCL8 is a prominent component of the SASP and is increased in chronic lung diseases. CXCL8 and the related CXCR1 induce senescence via the widely expressed receptor CXCR2 (Acosta et al, 2008). Both CXCL1 and CXCL8 are markedly increased in the sputum of patients with COPD (Traves et al, 2002), but CXCR2 antagonists are clinically ineffective in COPD, although long-term studies and effects on disease progression have not been reported (Chapman et al, 2009; Rennard et al, 2015). PAI-1 is another prominent SASP mediator that has been linked to cellular senescence, aging, and fibrosis (Vaughan et al, 2017). A null mutation of SERPINE1, the gene encoding PAI-1, has been associated with a prolonged lifespan (Khan et al, 2017). PAI-1 is increased in COPD and IPF (To et al, 2013; Schuliga et al, 2018) and thus may be a target for inhibition. Several classes of PAI-1 inhibitor have been developed but so far none have been approved for clinical use and none have been studied in lung disease (Sillen and Declerck, 2021).

Recent evidence suggests that IL-11, a proinflammatory and profibrotic cytokine belonging to the IL-6 family, may be an important mediator of cellular senescence through modulation of AMPK-mTOR pathways. Deletion of the IL-11 gene or its receptor prolongs lifespan in aging mice and an antibody to IL-11 delays the onset of age-related conditions such as cancer and frailty (Widjaja et al, 2024). Anti-IL-11 antibody is effective in preventing bleomycin-induced lung fibrosis in mice and antibodies to IL-11 are now in development for treating IPF and other fibrotic diseases (Ng et al, 2019). Because SASP is composed of multiple mediators and may vary in different cell types and inducers of senescence, it is unlikely that blocking single components will be effective and suggesting that a more useful approach would be to target the driving mechanisms, including NF-*κ*B, p38 MAPK, and JAK/STAT pathways.

#### 2 Nuclear factor-κB inhibitors

NF-*κ*B is a key regulator of the SASP, and its inhibition or genetic deletion reduces senescence, accelerated aging, and DNA damage in aged mice (Salminen et al, 2008; Tilstra et al, 2012). SIRT6 normally suppresses NF-*κ*B activation and its reduction with aging activates the SASP (Kawahara et al, 2009). A small molecule inhibitor of NF-*κ*B (SR12343) inhibits induced cellular senescence in vitro in mouse and human cells and reduces cellular senescence in murine models of aging (Zhang et al, 2021b). However, small molecule NF-*κ*B inhibitors are poorly tolerated in humans, so alternative approaches are needed (Ramadass et al, 2020).

#### 3 p38 mitogen-activated protein kinase inhibitors

P38 MAPK also drives the SASP response, mainly via NF-*κ*B activation, and a p38 MAPK inhibitor (SB203580) inhibits the secretion of many SASP mediators (Freund et al, 2011). SASP secretion from senescent human fibroblasts is markedly suppressed by selective p38 MAPK inhibitors (Alimbetov et al, 2016). Inhibiting MAPK-activated protein kinase-2, downstream of p38 MAPK, inhibits bleomycin-induced fibrosis in mice (Vittal et al, 2013). P38 MAPK inhibitors have also been poorly effective and have adverse effects in patients with COPD and are ineffective when given by inhalation (Millan et al, 2011).

#### 4 Janus-activated kinase inhibitors

The JAK/STAT pathway is activated in senescent cells, and inhibition of this pathway with a JAK1/2 inhibitor ruxolitinib reduces cellular senescence in vitro and in vivo in aged mice through suppression of SASP mediators (Xu et al, 2015). Ruxolitinib also reduces senescence in human fibroblasts in vitro and reduces accelerated aging phenotypes in mouse models of progeria (Griveau et al, 2020). Ruxolitinib also reduces aging phenotypes in mouse models of accelerated aging (Griveau et al, 2020). Although approved for use in hematological malignancies, JAK inhibitors have significant side effects after oral administration, including thrombocytopenia and anemia. The role of JAK-STAT pathways in the cellular senescence associated with chronic lung diseases has not been investigated. A JAK2 inhibitor fedratinib inhibits bleomycin-induced lung inflammation and fibrosis (Ruan et al, 2021). Inhaled pan-JAK inhibitors are now in clinical development and may be a promising alternative (Zak et al, 2019).

#### 5 Ataxia-telangiectasia mutated protein kinase inhibitors

ATM kinase is activated by DNA double-strand breaks as a consequence of oxidative stress and is an important orchestrator of the DDR, leading to p53 activation, cellular senescence, and SASP secretion. Several inhibitors of ATM kinase have been developed which have been shown to reduce cellular senescence (Kang et al, 2017; Kuk et al, 2019). The orally active inhibitor KU-55933 inhibits the development of cellular senescence and SASP mediator secretion in a mouse model of accelerated aging (Zhao et al, 2020) and inhibits cellular senescence in endothelial progenitor cells from patients with COPD (Paschalaki et al, 2013). Several ATM kinase inhibitors are currently in clinical development for the treatment of cancer (Priya et al, 2023).

### C Sirtuin activators

As discussed above, the reduction of SIRT1/6 in chronic lung diseases may accelerate lung aging by promoting cellular senescence. Calorie restriction in various species prolongs lifespan through increasing SIRT1. This has led to strategies to restore SIRT1/6 levels to normal, including chronic lung diseases (Li et al, 2023a).

#### 1 Resveratrol

Resveratrol (3, 5, 4'-trihydroxy-trans-stilbene) is a polyphenol found in the skin of red fruits and red wine, which has antiaging properties and extends lifespan in several species, through the direct or indirect activation of SIRT1 (Rogina and Tissenbaum, 2024). Resveratrol was first identified as an effective activator of yeast sirtuins (Howitz et al, 2003) and shown to prolong the life of mice on a high calorie diet (Baur et al, 2006). Resveratrol reduces oxidative stress and the SASP and in rats exposed to cigarette smoke (Wang et al, 2017) and inhaled resveratrol reduces lung aging in a mouse model of accelerated aging (Navarro et al, 2017) and reduces neutrophilic inflammation and inflammatory mediator release in rats exposed to lipopolysaccharide (Birrell et al, 2005). In vitro*,* resveratrol reduces SASP from alveolar macrophages of patients with COPD (Culpitt et al, 2003) and reduces senescence in endothelial progenitor cell from patients with COPD (Paschalaki et al, 2013). Many clinical trials have been conducted with resveratrol, and improvements in some age-related diseases, such as cardiovascular disease and type 2 diabetes, and a reduction in inflammatory biomarkers have been reported, although its efficacy is weak (Brown et al, 2024). In patients with COPD, treatment with resveratrol for 4 weeks did not increase SIRT1 or mitochondrial function in skeletal muscle (Beijers et al, 2020). The disappointing clinical benefits of resveratrol may be explained by its poor oral bioavailability and low potency. A related polyphenol, isorhapontigenin, has similar anti-inflammatory effects to resveratrol in airway epithelial cells and inhibits NF-*κ*B and PI3K signaling (Yeo et al, 2017). This compound is a constituent of a traditional Chinese herbal remedy for inflammatory diseases and has a greater oral bioavailability than resveratrol (Dai et al, 2018b).

#### 2 Sirtuin-activating compounds

Resveratrol not only has poor oral bioavailability but also has many other cellular actions, which led to a search for alternative sirtuin-activating compounds (STAC), such as SRT1720 and SRT2104. STACs are small molecules that are more potent and orally bioavailable (Dai et al, 2018a). STACs prolong the lifespan of normal and obese mice A (Mercken et al, 2014). SRT2172 reduces lung inflammatory cells, mediators, and MMP-9 in mice exposed to cigarette smoke who showed a reduction in lung SIRT1, with an increase in exercise performance and lung oxygenation (Nakamaru et al, 2009). SRT1720 protects against cigarette smoke-induced oxidative stress in mice through stimulating the antioxidant gene regulator FOXO3a (Yao et al, 2014). SRT2104 inhibits the development of cellular senescence in AT2 cells in a rat model of emphysema (Gu et al, 2020). SIRT1 is increased in the lungs of bleomycin-induced fibrosis of mice, but further activation of SIRT1 by SRT1070 inhibits the development of fibrosis by inhibiting the secretion of collagen from fibroblasts exposed to TGF-*β* (Zeng et al, 2017). SRT2104 was well tolerated in a phase 1 study in normal human volunteers (Hoffmann et al, 2013a). It improves psoriasis to a variable extent, with a reduction in SASP mediators, such as IL-17 and TGF-*β* (Krueger et al, 2015). The current clinical status of STACs in aging diseases is uncertain, and no clinical trials have been reported.

Other drugs may also increase the expression of SIRT1. The endogenous gas transmitter hydrogen sulfide (H_2_S) donor NaHS inhibits cigarette smoke-induced senescence in human airway epithelial cells through increasing the expression of SIRT1 (Guan et al, 2020). Curcumin restores the expression of SIRT1 in rats exposed to cigarette smoke in vivo and reverses the defect in autophagy (Tang and Ling, 2019).

### D NAD+ augmentation

Oxidized NAD^+^ is an endogenous activator of all sirtuins. NAD+ diminishes with aging and is reduced in several age-related diseases (Covarrubias et al, 2021). Because of the key role of NAD+ in senescence, inflammation, and immunity, several strategies have been used to boost NAD+ levels (Wu et al, 2024). NADH supplementation in mice reduces the secretion of SASP mediators following chronic cigarette smoke exposure (Slama et al, 2024). NAD^+^ levels may be boosted by nicotinamide mononucleotide, an intermediate precursor between nicotinamide and NAD, or by enzyme nicotinamide phosphoribosyltransferase, which scavenges NAD^+^ from nicotinamide, and can extend lifespan (Yaku et al, 2018). The NAD^+^ precursor nicotinamide riboside has been shown to boost NAD^+^ levels and reduce inflammatory biomarkers in a variety of clinical studies, but any effects on clinical outcomes are disappointing (Damgaard and Treebak, 2023). The flavonoids quercetin and apigenin inhibit the NAD^+^ase CD38, leading to an increase in NAD^+^ and thereby activating sirtuins (Hogan et al, 2019). Oral nicotinamide riboside given over 5 weeks increased plasma NAD^+^ in patients with COPD and reduced sputum CXCL8 concentrations, with some evidence of reduced epigenetic aging, although no direct evidence that cellular senescence is reduced (Norheim et al, 2024).

### E Targeting microRNAs

As discussed above, miR-34a inhibits SIRT1 and SIRT6 and is increased in peripheral lungs and epithelial cells of patients with COPD, and correlated with increased expression of senescence markers (Baker et al, 2016b). An antagomir of miR-34a, which blocks its action, restores SIRT1/6 in senescent small airway epithelial cells from patients with COPD, reduces markers of cellular senescence (p16, p21, p53), reduces SASP mediators (TNF-*α*, IL-1*β*, IL-6, CCL2, CXCL8, MMP9), and increases proliferation of senescent epithelial cells by reversing cell cycle arrest (Baker et al, 2016b). MiR-34a is released in EVs from senescent airway epithelial cells from patients with COPD and transmits senescence to normal recipient cells, with a decrease in SIRT1 and an increase in p16, p21, SA-*β*-gal, and SASP mediators (Devulder et al, 2025). Recipient cells loaded with miR-34a antagomir show no increase in senescence, demonstrating that inhibiting miR-34a may be a useful therapeutic strategy. MiR-570, which also inhibits SIRT1 (but not SIRT6) is increased in COPD peripheral lung and small airway epithelial cells. A miR-570 antagomir restores SIRT1, reduces senescence markers and SASP mediators, and reverses cell cycle arrest (Baker et al, 2019). This suggests that blocking specific miRNAs with antagomirs may result in restoration of reduced sirtuins and rejuvenate senescent cells in COPD. Antagomirs of specific miR-34a and miR-570 therefore have potential as antiaging therapies. However, until recently it has been difficult to treat cells with antagomirs because of poor cell penetration. Because miRNAs are taken up by cells via EVs, this suggests that these vesicles may be engineered to deliver antagomirs to target cells and could potentially be given by inhalation to ensure delivery of this cargo to airway epithelial cells (Zeng et al, 2022). Other miRNAs may have antiaging effects. The miR-302/367 cluster is associated with cell regeneration and targets p21, among several other targets (Li et al, 2023b). Recently EVs enriched with miR-302b have been shown to reverse cellular senescence in vitro and rejuvenate and prolong the lifespan of mice, with no detectable safety concerns over 24 months of administration (Bi et al, 2025).

### F Antioxidants

Oxidative stress, from cigarette smoke or air pollution, and from activated inflammatory and structural cells in the lung, is a major driver of cellular senescence in COPD (Barnes, 2022) and from inflammatory and structural cells in IPF and other chronic lung diseases (Estornut et al, 2021). This suggests that antioxidants should be effective inhibitors of cellular senescence and SASP mediator release in chronic lung diseases. Unfortunately, currently available antioxidants, such as *N*-acetylcysteine, carbocysteine, and erdosteine, which are also mucolytics, are poorly effective as antioxidants as a high level of oxidative stress in the lungs inactivates these drugs, suggesting that more potent antioxidants are needed (Barnes, 2020). Although oxidative stress is considered to be an important driving mechanism in many diseases, it has proved challenging to develop more effective and safer antioxidants.

#### 1 Nuclear factor erythroid 2-related factor 2 activators

The transcription factor nuclear factor erythroid 2-related factor 2 (Nrf2) regulates multiple antioxidant genes in order to protect cells against oxidative stress (Cho et al, 2006; Wang et al, 2022). However, Nrf2 is defective in COPD and IPF, resulting in increased oxidative stress to drive further cellular senescence (Lee et al, 2021a). A synthetic triterpenoid Nrf2 activator bardoxolone methyl is effective in a cigarette smoke-exposed mouse model of COPD (Sussan et al, 2009), but a phase 3 clinical trial in renal disease was terminated due to adverse effects and increased mortality (Rossing, 2013). Small molecule Nrf2 activators, such as sulforaphane and bardoxolone, are nonspecific and toxic, prompting a search for new activators (Cuadrado et al, 2019). A clinical trial of sulforaphane in patients with COPD was negative, although target engagement was not demonstrated (Wise et al, 2016).

Nrf2 is normally activated by the proteolysis of Kelch-like ECH-associated protein 1 (KEAP1) to which it is bound in the cytoplasm, thus releasing Nrf2 to enter the nucleus, where it binds to antioxidant response elements in the promoter region of antioxidant genes. Several electrophilic modifiers of the sensor KEAP1 are in clinical development but a major problem is specificity (Dinkova-Kostova and Copple, 2023). One such compound dimethyl fumarate (BG-12) has already been approved for use in multiple sclerosis and psoriasis but has not been studied in chronic lung disease. In a mouse model of oxidative stress due to exposure to inhaled diesel exhaust particulates, dimethyl fumarate reduced KEAP1, oxidative/nitrosative stress, and reduced inflammatory mediators, although markers of senescence were not reported (Cattani-Cavalieri et al, 2020). A microparticulate formulation of BG-12 has been developed for aerosol administration (Muralidharan et al, 2016), but no clinical studies have been reported. Inhaled dimethyl fumarate was more effective than orally administered drug in reducing bleomycin-induced lung fibrosis in mice (Kato et al, 2022).

In order to increase specificity, several peptide and small molecule drugs that inhibit the protein–protein interaction between Nrf2 and KEAP1 are in development. Several small molecules with a high affinity for KEAP-1 have been developed although these drugs appear less potent in cell systems (Dinkova-Kostova and Copple, 2023). Bach1 (BTB and CNC homology-1) is an endogenous inhibitor of Nrf2 in some cell types, so another strategy is the development of Bach1 inhibitors to increase activation of Nrf2 (Mafra et al, 2022; Hushpulian et al, 2024).

#### 2 NADPH oxidase inhibitors

NADPH oxidase (Nox) is a membrane-bound complex that is the major source of ROS in COPD through the generation of superoxide anions. Several isoforms of the catalytic component of Nox exist, including Nox1-5 and the dual oxidases Duox1 and 2 (Elbatreek et al, 2021). Nox may also contribute to increased oxidative stress in COPD and IPF (Schiffers et al, 2021). Selective Nox inhibitors have now been developed, and clinical trials are in progress (Chocry and Leloup, 2020; Cipriano et al, 2023). Apocynin, a nonselective Nox inhibitor, reduces inflammatory cytokines and chemokines in bronchoalveolar lavage fluid in cigarette-smoke exposed mice (Oostwoud et al, 2016). Nebulized apocynin reduces exhaled H_2_O_2_ and nitrite concentrations in exhaled breath condensate of patients with COPD, but no clinical parameters are reported (Stefanska et al, 2012). Setanaxib (GKT137831) is a dual Nox1/4 inhibitor which attenuates acute lung injury induced by ischemia-reperfusion injury but has not been studied in models of chronic lung disease (Cui et al, 2018). Nox4 has been implicated in the mechanisms of pulmonary fibrosis, suggesting that Nox4 inhibitors may be useful in IPF (Ghatak et al, 2017). Setanaxib inhibits TGF-*β* induced activation of COPD fibroblasts so may be indicated in IPF (Huang et al, 2017).

#### 3 Mitochondrial antioxidants

As discussed above, mitochondrial dysfunction is one of the hallmarks of aging and a key feature of chronic lung diseases, such as COPD and IPF cells (Birch et al, 2018; Cloonan et al, 2020). Mitochondrial dysfunction is closely linked to cell senescence through activation of p53, which inhibits PPARγ coactivator 1*α*, a transcriptional coactivator that is a key regulator of mitochondrial function and regulated by SIRT1. Damaged and abnormal mitochondria may be a major source of ROS in COPD (Wiegman et al, 2015; Belchamber et al, 2019). Senotherapies should reduce mROS through improvement of mitochondrial function. Several mitochondria-targeted antioxidants, based on the structure of ubiquinone, are concentrated 50- to 100-fold in mitochondria and are more effective in animal models of aging than conventional antioxidants (Kolosova et al, 2012; Murphy, 2014). Mitochondria-targeted antioxidants include mitoQ, mito-TEMPO, pyrroloquinoline quinone, and SkQ1, which are now in clinical trials for other age-related diseases. Cigarette smoke extract causes mitochondrial dysfunction in human airway epithelial cells in vitro and release of mROS, which is inhibited by mito-TEMPO (Hara et al, 2013). In a mouse model of chronic oxidative stress, mitoQ treatment reduces airway hyperresponsiveness, neutrophilic inflammation, and lung inflammatory mediators (Wiegman et al, 2015). There is a good rationale for clinical studies with mitochondria-targeted antioxidants in COPD and other chronic lung disease. No clinical studies in chronic lung disease have been reported although several trials are underway (Fairley et al, 2023).

#### 4 H_2_S donors

Endogenous H_2_S protects against cellular senescence, and mitochondria-targeted H_2_S donors, such as AP39 and AP123, reduce senescence and SASP in senescent primary endothelial cells (Gero et al, 2016; Latorre et al, 2018). These H_2_S donors are effective in some modes of accelerated lung aging and reduce cellular senescence (Pacitti et al, 2021).

## VII Senolytic drugs

**Fig. 7:**
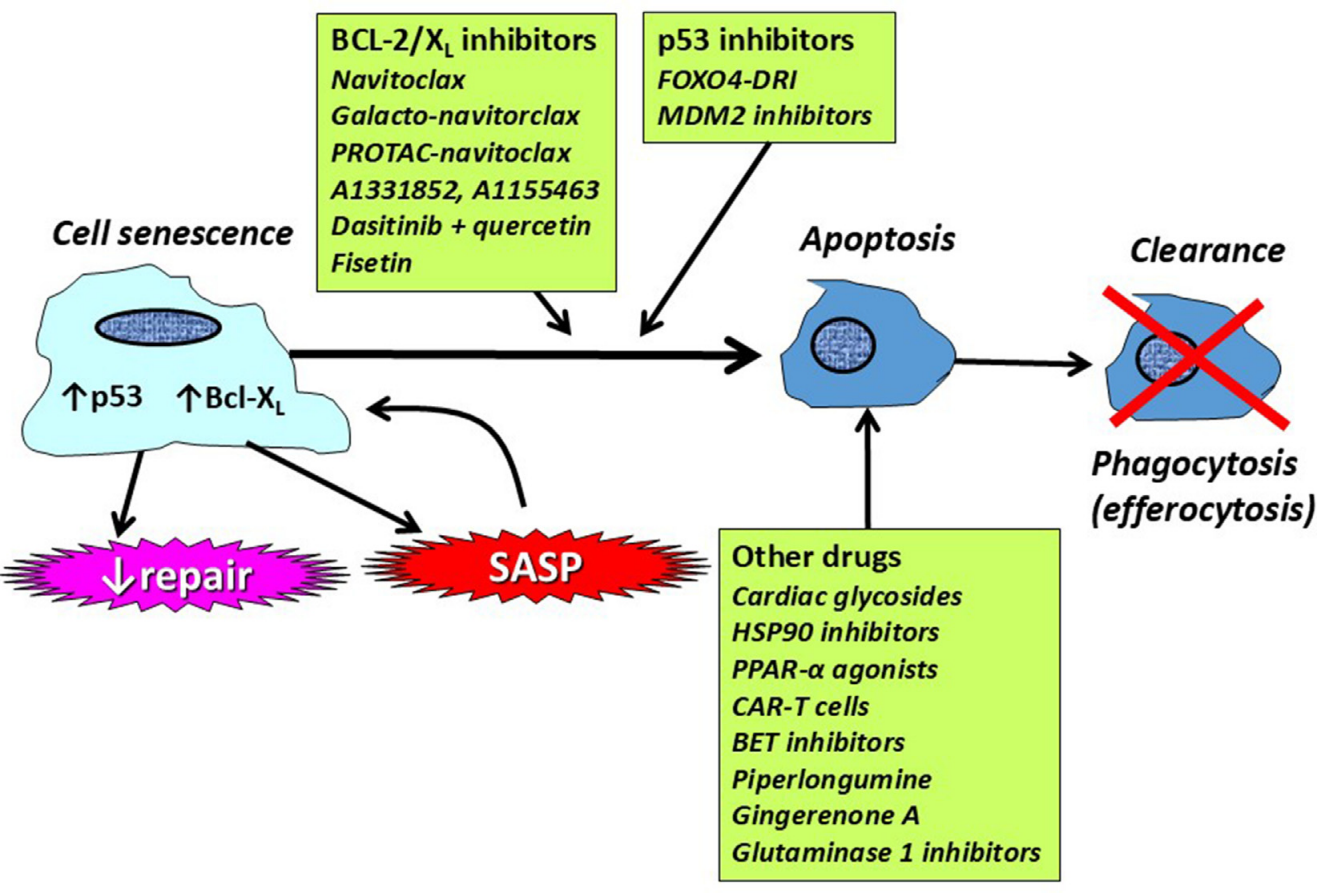
Senolytic therapies. Senolytics drive the senescent cell in cell cycle arrest toward apoptosis and subsequent clearance from the tissue by efferocytosis. Senolytic therapies include inhibitors of the antiapoptotic Bcl family and inhibitors of p53, and several other classes of drug.

Senolytic drugs remove senescent cells without damaging normal proliferating cells by targeting specific vulnerabilities of senescent cells, by inducing apoptosis, or potentially other forms of cell death. Apoptotic cells may then be cleared from the lungs by phagocytosis (efferocytosis). This may be achieved by targeting cell survival pathways, such as Bcl2 family members, p53 and p21 (Zhang et al, 2023). Several classes of senolytic drug have now been identified and some are in clinical development for the treatment of age-related diseases (Fig. 7; Table 2) (Chaib et al, 2022). Novel senolytic molecules have been identified by high-throughput in vivo screening (Lee et al, 2024).

**Table 2:** Senolytic drugs that have been studied in lung disease in vitro and in vivo

Target	Drug	In Vitro	In Vivo
Bcl-2	Navitoclax	+	+
(Zhu et al, 2016; van der Feen et al, 2020; Cooley et al, 2023)			
Bcl-XL	A-1331852, A-1155463	+	
(Zhu et al, 2016)			
Bcl-2/PI3K	Dasitinib + quercetin	+	+
(Lehmann et al, 2017; Schafer et al, 2017; Justice et al, 2019; Nambiar et al, 2023)			
Bcl-2/PI3K	Fisetin	+	+
(Camell et al, 2021)			
p53	FOXO-4DRI	+	+
(Baar et al, 2017; Han et al, 2022; Born et al, 2023; Liu et al, 2023b)			
Cardiac glycoside	Ouabain, digoxin	+	+
(Guerrero et al, 2019; Triana-Martínez et al, 2019; Martin et al, 2020)			
HSP90	Alvespimicin, tanespimicin, XL88	+	+
(Fuhrmann-Stroissnigg et al, 2017; Lee et al, 2024)			
PPARα	Gemfibozil, fenofibrate	+	+
(Lakatos et al, 2007; Bodas et al, 2017)			
COP1	DD86481,MP1320	+	+
(McHugh et al, 2023)			

### A Dasatinib-quercetin

The combination of dasatinib, a tyrosine kinase inhibitor approved for the treatment of chronic myeloid leukemia, and the dietary flavonoid quercetin (D+Q) induces senolysis by inhibiting Bcl2 and also inhibits PI3K signaling. D+Q reduces senescent cells, p16 expression, and the SASP response, extends lifespan, and reduces frailty in elderly mice (Xu et al, 2018). D+Q has shown efficacy in several animal models of age-related diseases and several clinical trials are underway (Chaib et al, 2022). D+Q is effective in clearing senescent cells from the lungs in bleomycin-induced fibrosis and improves pulmonary function in mice (Lehmann et al, 2017; Schafer et al, 2017). Orally administered D+Q (3 days a week over 3 weeks) was well tolerated in patients with IPF (Justice et al, 2019). In elderly patients with diabetic kidney disease the same treatment given over 3 days reduces senescent cells in the skin and SASP proteins (IL-1, IL-6, and MMP-9) in the blood after 3 weeks and is well tolerated (Hickson et al, 2019). In a small randomized controlled trial of 12 patients with IPF, D+Q was given for 3 days/week over a 3-week period. There was an increase in adverse effects, including nausea, fatigue, and headaches, but no withdrawals form the study (Nambiar et al, 2023). There was no improvement in symptoms or lung function, but this is expected with the short duration of the study and the small numbers included. Clinical trials of D+Q for longer treatment periods in IPF are underway (NCT02874989)

### A Bcl-2 family inhibitors

Senescent cells upregulate the antiapoptotic Bcl-2 family members, including Bcl-2, Bcl-X_L_, and Bcl-w to prevent apoptosis and promote senescent cell survival. There proteins have a shared BH3 region that is targeted by several inhibitory compounds. ABT-732 was the first inhibitor of Bcl-2 family members shown to have senolytic activity and reduced senescent cells in the lungs and skin, with an increase in stem cell proliferation (Yosef et al, 2016). Because of poor oral bioavailability a derivative ABT-263 (navitoclax) was developed as a cancer therapy and shown to have similar senolytic effects (Zhu et al, 2016). However, a major problem with navitoclax is that it may cause thrombocytopenia and neutropenia. Venetoclax is a more selective inhibitor of Bcl-2 than Bcl-X_L_ and has less effect on platelets, so is approved for the treatment of hematological malignancies, although it has less senolytic efficacy than navitoclax (Fowler-Shorten et al, 2024). Navitoclax induces apoptosis in senescent mouse lung fibroblasts and reduces fibrosis in a mouse model of IPF (Cooley et al, 2023). Navitoclax also targets senescent pulmonary vascular endothelial cells and reverses pulmonary artery remodeling and blood pressure in a rat model of PAH (van der Feen et al, 2020). Inhibition of Bcl-X_L_ appears to be critical for senolytic effects in many cell types and the more selective Bcl-X_L_ inhibitors A-1331852 and A-1155463 (Schafer et al, 2017; Justice et al, 2019) are effective senolytics in senescent human endothelial cells and lung fibroblasts (Zhu et al, 2016). A Bcl-xL inhibitor foselutoclax (UBX1325) has been studied in clinical trials in diabetic macular edema and shown to be well tolerated with evidence of disease modification (Crespo-Garcia et al, 2024). This drug also improves retinal function in patients with age-related macular degeneration (Macha et al, 2024). Local administration may also abrogate the adverse effects on platelets. Intra-articular injection of navitoclax was effective in removing senescent cells in a rat model of osteoarthritis (Yang et al, 2020), suggesting that inhaled administration might be effective in chronic lung diseases.

Another strategy to reduce adverse effects of Bcl-2 inhibitors and other senolytics is conjugation with galactose so that the drug is selectively released in senescent cells which have SA-*β*-gal activity. Galactose-navitoclax has greater senolytic activity and less effects on platelets in mice compared to navitoclax, so may be a promising strategy (González-Gualda et al, 2020). A further strategy is to link navitoclax to proteolysis-targeting *c*himeras (PROTAC), which are bifunctional molecules comprising a ligand (navitoclax) linked to a ligand that recruits an E3 ubiquitin ligase, resulting in the ubiquitination and subsequent destruction of the target protein (Bcl-2 family) via the ubiquitin proteasome system. This results in more selective targeting and longer duration of action with reduced toxicity. PROTAC-navitoclax is effective in removing senescent cells from aged mice with less thrombocytopenia than navitoclax (He et al, 2020b). Several PROTAC linked Bcl-2 and Bcl-X_L_ inhibitors have now been developed, which have longer duration of action and less hematological adverse effects than navitoclax (Nayak et al, 2024).

Myeloid cell leukaemia-1 (Mcl-1) is another member of the Bcl-2 family that may inhibit apoptosis in senescent cells, Mcl-1 is upregulated in senescent cancer cells and the Mcl-1 inhibitor S63845 eliminates senescent cancer cells more effectively than navitoclax (Troiani et al, 2022). Inhibition of Mcl-1 enhances the senolytic effects of other Bcl-2 inhibitors (MacDonald et al, 2025). However, studies have not been reported in noncancerous senescent cells.

### C p53 inhibitors

Replicative cellular senescence involves the activation of the transcription factor p53, which activates p21 promoting cell cycle arrest. Inhibition of p53 induces apoptosis and therefore senolysis.

#### 1 Forkhead box O 4 D-retro-inverso

FOXO4 is increased in senescent cells and binds to p53 in the nucleus to prevent its translocation to the cytoplasm. A cell penetrant peptide, a D-retro-inverso form of FOXO4 (FOXO4-DRI), prevents this interaction of FOXO4 with p53, thus leading to cytoplasmic translocation and apoptosis and removal of senescent cells; this reduces age-related organ failure and frailty in mice (Baar et al, 2017). FOXO4-DRI eliminates TGF*β*-induced myofibroblasts in vitro and prevents bleomycin-induced pulmonary fibrosis and increases AT2 cell regeneration in mice (Han et al, 2022; Liu et al, 2023b). However, in a mouse model of hypoxia-induced PAH, in which pulmonary vascular endothelial cells are senescent, FOXO4-DRI paradoxically increases pulmonary hypertension, with the elimination of senescent endothelial cells (Born et al, 2023), which is potentially an example of the heterogeneous function of senescent cells in different contexts.

#### 2 Mouse double minute 2 inhibitors

Although p53 inhibition may promote senolysis, increasing p53 may also have a senolytic effect in some cell types and with some inducers of senescence. Mouse double minute 2 (MDM2) is an E3 ubiquitin ligase that targets p53 for proteolysis and acts as a critical inhibitor of p53-induced senescence (Huang et al, 2024). Several small molecule drugs that inhibit the protein-protein interaction between MDM2 and p53, and PROTAC drugs that target MDM2 are in development for cancer and have been shown to have senolytic effects (Fallatah et al, 2023). Inhibition of MDM2-p53 interaction with a small molecule inhibitor reduces senescent cells and improves skeletal muscle function in aged mice (Nolt et al, 2024). A potent MDM2 inhibitor (RG-7112) reduces senescent cells and SASP mediators in human intervertebral discs in vitro (Cherif et al, 2020), but no studies in chronic lung disease have been reported. Ubiquitin-specific peptidase 7 deubiquitinates MDM2 to increase its expression. Inhibition of ubiquitin-specific peptidase 7 with small molecules such as P5091 and P2207 selectively eliminates senescent cells by reducing MDM2 leading to increased p53 and inhibition of Bcl-X_L_ (He et al, 2020a; Zhang et al, 2024).

### D Fisetin

A dietary flavonoid fisetin (3,3′,4′,7-tetrahydroxyflavone) is found in many fruit and vegetables, particularly strawberries, and is an effective senolytic drug comparable to D+Q, and more effective than quercetin alone (Zhu et al, 2017). Fisetin inhibits Bcl-2 and Bcl-X_L_ and activates caspase-3, which promotes apoptosis, as well as inhibiting PI3K-mTOR signaling and having antioxidant effects, all of which may reduce the development of cellular senescence (Chen et al, 2002; Verma et al, 2017; Wang et al, 2018a). In mouse models of accelerated aging fisetin reduces senescent cells, even when given to aged animals, as well as extending the lifespan and healthspan of normal mice (Yousefzadeh et al, 2018). Fisetin improves survival in old mice infected with a *β*-coronavirus similar to SARS-CoV-2, with a reduction in senescent cells that drive the systemic inflammatory response (Camell et al, 2021). Fisetin is well tolerated even in high doses in animals (Ravula et al, 2021) and after prolonged administration in humans (Wissler Gerdes et al, 2021). Currently, fisetin is in clinical trials for several age-related diseases, including IPF (NCT05593588).

### E Cardiac glycosides

Screening studies have identified several cardiac glycosides, including ouabain, digoxin, and bufalin, as senolytic drugs (Martin et al, 2020). Ouabain, digoxin, and digitoxin have senolytic effects in a wide variety of cell types and senescence inducers. In the bleomycin model of lung fibrosis intermittent treatment with digoxin reduces markers of senescence and depletes senescent cells. Intermittent ouabain reduces fibrosis and also removes human senescent fibroblasts transplanted into the lungs of immunodeficient mice (Triana-Martínez et al, 2019). Similarly, ouabain induces apoptosis various senescent cells and removes senescent cells from the lungs of irradiated and aging mice, with a reduction in SASP mediators and a reduction in frailty (Guerrero et al, 2019). The mechanisms of senolytic action are due to inhibition of the Na^+^/K^+^ ATPase pump, which promotes apoptosis in senescent cells, but not normal cells, by causing further depolarization and acidification in senescent cells, making the inhibition of this pump more detrimental (Triana-Martínez et al, 2019). These findings suggest that cardiac glycosides such as digoxin which are in clinical use for the treatment of atrial fibrillation and heart failure may be repurposed as senolytic drugs as the concentrations required for senolysis are similar to those achieved in clinical practice (20–30 nM) (Lee et al, 2023).

### F Heat shock protein 90 inhibitors

Heat shock protein 90 (HSP90) acts as a chaperone protein that stabilizes many intracellular proteins, and several inhibitors of HSP90 that have been developed for cancer therapy, including geldanamycin, alvespimicin (17-DMAG), and tanespimicin (17-AAG), have been shown to have senolytic effects in an aged mouse model with extension of healthspan, human lung fibroblasts, and human umbilical vein endothelial cells (Fuhrmann-Stroissnigg et al, 2017). The mechanism for their senolytic action is uncertain, but HSP90 inhibits apoptosis via stabilization of AKT which inhibits pro-apoptotic pathways, inhibiting telomerase and inhibiting the DDR (Dutta Gupta and Pan, 2020). Using high-throughput screening a novel HSP90 inhibitor XL888 was found to eliminate senescent fibroblasts in bleomycin-treated mice and in precision-cut lung slices from patients with IPF (Lee et al, 2024).

### G Peroxisome proliferator activated receptor α activators

PPAR*α* agonists, such as the fibrates fenofibrate and gemfibozil, are currently used as lipid lowering therapies but also target TFEB, the key regulator of autophagy. Gemfibrozil protects human airway epithelial cells from autophagy induced by cigarette smoke exposure in vitro and TFEB knockout mice show impaired autophagy and increased cellular senescence, which is prevented by gemfibozil therapy (Bodas et al, 2017). Gemfibozil also protects against the impaired autophagy induced by cigarette smoke exposure in mice and reduces senescence markers and emphysema. Fenofibrate reduces senescence in human chondrocytes in vitro through promotion of autophagy and apoptosis (Nogueira-Recalde et al, 2019). Although there are no clinical trials of fibrates on COPD outcomes, retrospective studies have shown a reduced risk for COPD in hyperlipidemic patients on fibrates (Lei et al, 2020). A PPAR*α* agonist is also effective in a bleomycin model of IPF in mice (Lakatos et al, 2007). Because fibrates are well tolerated, they may be repurposed to treat age-related lung diseases in future studies.

### H Harnessing the immune system

Chimeric antigen receptor (CAR) T-cells targeting the senescence-associated protein urokinase plasminogen activator receptor that may be expressed on the surface of senescent cells eliminates senescent cells in vitro and in vivo (Amor et al, 2020). A single administration of anti- urokinase plasminogen activator receptor CAR-T cells has a long-lasting effect and delays aging-related pathologies in mice (Amor et al, 2024). CAR-T cells that target the receptor for natural killer group 2 member D ligands, which are also increased in senescent cells, are also effective in eliminating senescent cells in vitro and in mice and nonhuman primates in vivo without any obvious adverse effects (Yang et al, 2023). Anti-natural killer group 2 member D ligand receptor CAR-T cells eliminate senescent fibroblasts with senescence induced by oxidative stress, so this may be a promising approach as a long-term senolytic therapy in chronic lung diseases (Deng et al, 2024).

Another approach is to enhance the removal of senescent cells from tissue by natural killer cells. Screening with siRNAs has identified that inhibition of the transcriptional regulator gene *SMARCA4* (that encodes for a chromatin remodelling switch/sucrose non-fermentable complex component) activates the cyclic GMP-AMP synthase/stimulator of interferon gene to enhance NK-dependent removal of senescent cells (Reen et al, 2025).

### I Other senolytics

#### 1 Bromodomain and extra-terminal domain inhibitors

High-throughput screening has identified a bromodomain and extra-terminal domain (BET) family protein inhibitors, such as JQ1, as a potent senolytic which eliminates senescent cells by limiting DNA damage and promoting autophagy in senescent cells, irrespective of the mechanisms of senescence (Wakita et al, 2020). ARV825 is a PROTAC-linked BRD4 degrader that is particularly effective as a senolytic (Wakita et al, 2020). One effect of BET inhibitors is a reduction in SASP release and BRD4 inhibitors suppress the secretion of SASP mediators, such as IL-1*β* and CXCL8, from airway epithelial cells from patients with COPD (Khan et al, 2014). Several BET inhibitors are now in clinical development.

#### 2 Piperlongumine

Piperlongumine is a naturally occurring alkaloid from the roots of long pepper plants and was discovered to have senolytic activity in screening assays and eliminates senescent cells after different cellular stresses (Wang et al, 2016). Several more potent analogues with greater senolytic activity are in development, although their mechanisms of action remain uncertain (Wang et al, 2016; Liu et al, 2018).

#### 3 Gingerenone A

An extract of ginger was found to have senolytic effects on senescent human fibroblasts and the active compound identified as gingerenone A, with similar efficacy to D+Q, though inhibition of Bcl-X_L_ and increased caspase-3 (Moaddel et al, 2022).

#### 4 Glutaminase 1 inhibitors

Glutaminase 1 (GLS-1) was discovered, using short hairpin RNA libraries, to be a gene essential to maintain senescence in human cells and is highly expressed in aged lungs. Increased GLS-1 in response to the low pH in senescent cells induces the formation of ammonia, which neutralizes pH and prolongs survival. Inhibiting GLS-1-with BPTES (bis-2-[5-phenylacetamido-1,3,4-thiadiazol-2-yl]ethyl sulfide] and other selective inhibitors depletes senescent human fibroblasts and eliminates senescent cells associated with reduced lung fibrosis in aged mice (Johmura et al, 2021).

#### 5 cFLIP inhibitors

A CRISPR-based senolytic screen identified cFLIP as inhibitor of death receptor (DR)-mediated apoptosis in senescent cancer cells. Agonists of DR4 and DR5 sensitize senescent cancer cells to apoptotic death, without affecting healthy cells. This suggests that cFLIP inhibitors may act as senolytic therapy, although this has not been studied in noncancerous cells.

#### 6 N-myristoyltransferase inhibitors

RNAi screening of senescent cells identified coatomer complex I (COPI) vesicle formation as a vulnerability in senescent cells, which is inhibited by N-myristoyltransferase inhibitors, such as DD86481 and IMP1320. These N-myristoyltransferase inhibitors showed senolytic effects in vitro and in bleomycin-induced lung fibrosis in mice in vivo, without significant toxicity (McHugh et al, 2023).

## VIII Clinical issues and safety

In conducting clinical trials with senotherapies, it is important to consider their impact on a variety of age-related clinical outcome measurements and to carefully study the cells targeted by each drug. There is a need for better markers of specific senescent cell phenotypes that are involved in chronic lung diseases, so that more selective senomorphics and senolytics can be developed in the future (Romashkan et al, 2021).

### A Safety

An important consideration is safety of these treatments, which may be given to elderly individuals, who often have other age-related diseases, such as heart disease, metabolic diseases, and chronic kidney disease, and are likely to already be treated with several other drugs that could interact with senotherapies.

Safety after long-term administration is an important issue with senomorphic drugs that may be given on a daily basis over many years to often elderly and frail patients. Existing drugs, such as metformin, rapalogs, and resveratrol, may be repurposed, so the clinical safety of these drugs is well established, although these drugs may have several additional mechanisms of action. Metformin is well tolerated in patients with COPD and may therefore be a promising senomorphic treatment (Hitchings et al, 2015; Polverino et al, 2021). Senolytics may be effective with intermittent treatment using a “hit-and-run” strategy to decrease the senescent cell burden with the next treatment given several weeks later as new senescent cells accumulate. In mice with age-related osteoporosis D+Q is effective in restoring bone if given every few weeks, although the elimination half-life is only about 11 hours and senescent cells reaccumulate slowly (Chaib et al, 2022). In a pilot clinical study D+Q was given as a single dose and senescent cells were reduced in the skin 3 weeks later (Hickson et al, 2019). The dose and dosing frequency of senolytic treatments remains to be determined and may depend on the disease under treatment and the stage of the disease. Nevertheless, intermittent therapy means that side effects are less of an issue than for chronic administrations, and even potentially severe side effects such as thrombocytopenia with navitoclax could be monitored and treated if necessary. Only limited clinical studies have been undertaken with senolytics, but D+Q in small open studies has been well tolerated, although the duration of study is too short to assess long-term safety (Hickson et al, 2019; Justice et al, 2019; Nambiar et al, 2023). Fisetin is well tolerated in humans, and several clinical trials in age-related diseases including IPF are currently underway.

#### 1 Cancer risk

Cellular senescence may have evolved as a mechanism to prevent the development of cancers, so that senotherapies may theoretically increase cancer risk. Several human cancers are associated with the development of cellular senescence, and this may have a protective role against tumor progression. Anticancer therapies, including cytotoxic drugs, and irradiation that activates the DDR, are also well recognized as inducers of cellular senescence and this may reduce resilience of anticancer therapies and promote cancer recurrence (Calcinotto et al, 2019). However, many studies have shown that cellular senescence is associated with worse clinical outcomes and tumor progression. The SASP, which contains various growth factors, associated with cellular senescence may promote the development of cancers and the switch from precancer to malignancy and may account for the increasing prevalence of certain cancers with age. There is increasing evidence that senomorphics and senolytics may target several types of cancer and may improve responses to different anticancer therapies (Wyld et al, 2020). Senolytic therapies have shown efficacy as anticancer therapies in vitro and in vivo in animal models. For example, fisetin induces autophagy in a mouse model of pancreatic cancer (Jia et al, 2019). PROTAC-navitoclax is highly effective against human small cell lung cancer cells in vitro (Khan et al, 2024).

#### 2 Wound healing

Acute senescence is important for acute tissue repair and reduces excessive fibrosis during cell proliferation (Wilkinson and Hardman, 2020). Senescent cells promote wound healing through the secretion of growth factors, such as PDGF-AA; ablation of senescent cells impairs wound healing in the skin of mice (Demaria et al, 2014). However, chronic senescence is detrimental to wound healing as the SASP is proinflammatory and may account for delayed wound healing in the elderly (Wilkinson and Hardman, 2020), so it is unlikely that senotherapies will have a significant effect on wound healing after injury or surgery. Indeed, senotherapies are now considered to be a potential therapy for poorly healing wounds. In one study, elimination of senescent endothelial cells by the senolytic FOXO4-DRI increases pulmonary hypertension in mice (Born et al, 2023), suggesting that there may be adverse effects of senolytics on the vasculature. However, this has not been reported in studies of senolytic drugs in models of cardiovascular disease (Tian et al, 2024).

#### 3 Combinations

One strategy to increase efficacy and reduce side effects involves combining different senomorphics and senolytic drugs, which may have additive or synergistic effects so that lower doses may be used with less risk of adverse effects. For example, mTOR inhibitors increase the senolytic activity of navitoclax (Xu et al, 2022).

#### 4 Galactose prodrugs

Linking a senolytic drug to galactose creates a prodrug that takes advantage of the increase SA-*β*-gal activity in senescent cells to release the active senolytic drug selectively within senescent but not normal cells to reduce toxicity. This strategy has been successful with navitoclax, as discussed above (González-Gualda et al, 2020). A prodrug with galactose linked to the cytotoxic drug gemcitabine, which eliminates human senescent cells in vitro, irrespective of the mechanism of senescence or cell type (Cai et al, 2020). This prodrug is also effective in removing SA-*β*-gal positive cells from tissues in aging mice and improving age- and senescence-related gene signatures. Similarly, a galactose-linked cytotoxic drug duocarmycin was effective in eliminating SA-*β*-gal positive cells in mice (Guerrero et al, 2020).

A similar approach involves encapsulation of a drug with galactose oligosaccharides. Gal encapsulated cytotoxic drugs, such as palbociclib, remove senescent cells from mice in vitro and reduce collagen deposition and restore lung function in the bleomycin IPF mouse model in vivo (Muñoz-Espín et al, 2018).

#### 5 Proteolysis targeting chimeras

As discussed above, a PROTAC-navitoclax (PZ15227) is effective in removing senescent cells from aged mice with less thrombocytopenia than navitoclax (He et al, 2020b). Several PROTAC linked Bcl-2 and Bcl-X_L_ inhibitors have now been developed, which have longer duration of action and less hematological adverse effects than navitoclax (Nayak et al, 2024). The PROTAC approach may also be used to more specifically target other classes of senolytic drug in the future.

### B Route of administration

Although systemic administration is the most likely mode of administration and the route of delivery used in current clinical trials of senotherapies for other disease, it may be possible to improve safety by local delivery to the lung by inhalation. This strategy has been used to improve the safety of bronchodilators and corticosteroids in obstructive lung diseases, as systemic administration of these drugs is associated with side effects. However, it may be more difficult to target alveolar senescence by inhalation as it is difficult to retain drugs in the alveolar compartment as they rapidly enter the circulation. Local administration of navitoclax via intra-articular injection appears to be effective in removing senescent cells from joints in a rat model of osteoarthritis (Yang et al, 2020), suggesting that the inhaled route might be effective in chronic lung diseases. However, systemic administration may also be preferred in COPD as most patients have comorbidities that are also age-related diseases with an increase in senescent cells.

### C Heterogeneity

An important issue in senotherapeutics is the heterogeneity in senescent cell phenotypes, according to the cell type, the stimulus inducing senescence, the composition of the SASP, and the stage of senescence and the disease itself. This implies that senotherapies may have different effects depending on the context, with senescent cells and disease type responding differently to these drugs. This mandates careful characterization of senescent cells involved in different lung diseases as senotherapies that are effective in one disease may not less effective in another disease.

### D Biomarkers

In order to assess suitability of patients for senotherapy and to monitor their effects, it is necessary to use readily available and validated biomarkers, either in blood, sputum, or exhaled breath samples. Biomarkers of senescence, such as SA-*β*-gal, telomere length, γH2AX, p21, p16, and SAP mediators, may be measured in either cells or fluids and more than one marker should be used to confirm cellular senescence (Gorgoulis et al, 2019). In addition, multiomics approaches may be used to demonstrate activation of senescence pathways (Muthamil et al, 2024). Epigenetic clocks of aging have been devised from analysis of DNA methylation patterns, although there is wide biological and technical variability and a lack of specificity for particular age-related diseases (Higgins-Chen et al, 2022). A machine-learning approach to lung aging based on DNA methylation has recently been developed which may be more precise (R. Sehgal et al, preprint, DOI: https://doi.org/10.1101/2023.07.13.548904).

However, there is still no consensus on which biomarkers of aging should be validated before being used in clinical studies to monitor the effects of senotherapies (Moqri et al, 2024). This is of great importance for future clinical trials of senotherapies.

### E Future perspectives

The in vitro data on human lung cells and in vivo studies in animal models of chronic lung disease strongly support the potential of senotherapies to reduce disease progression and even reversal of disease (Barnes, 2023; Sugimoto, 2024; Wang et al, 2024). There is growing evidence that senotherapies are effective in many mouse models of age-related diseases, including chronic lung diseases, but there are few studies in human disease (McHugh et al, 2025). In chronic lung diseases, senotherapies should lead to a reduction in chronic neutrophilic inflammation, improved immune function, reduced fibrosis, and, potentially, regeneration of damaged cells. However, clinical studies of senotherapies in lung diseases are challenging as disease progression in most chronic lung diseases is slow, so that long-term studies will be needed to demonstrate meaningful clinical benefits. It is predicted that senotherapies may not only reduce disease progression and mortality, but may also reduce age-related comorbidities, which are commonly seen in COPD and IPF. Inhibiting SASP mediators may also lead to improved quality of life and reduced symptom burden, with reduced exacerbations if the chronic disease.

#### 1 Monitoring cellular senescence

In order to study the clinical effects of senotherapies, it is important to develop useful biomarkers of cellular senescence, as discussed above. Although biopsies may be used to measure senescent cell numbers, airway biopsies are invasive and difficult to repeat and do not sample small airways or lung parenchyma, which are the major sides of disease in COPD and IPF, respectively. Measuring SASP mediators in blood, sputum, or bronchoalveolar lavage is feasible but may not be specific for cellular senescence because the same inflammatory mediators are produced in response to other stimuli. PAI-1 appears to be more specific for cellular senesce and is increased in sputum of patients with COPD (To et al, 2013). More useful would be markers of senescence in cells obtained from patients with chronic lung disease, such as macrophages and shed epithelial cells in induced sputum, although cell numbers may be low. New techniques are in development for measuring protein and miRNA expression in single airway cells that may make this feasible in the future (Ho et al, 2023). Because senescence may affect extrapulmonary tissues, biopsies from remote organs, such as skin and the nose, may be used to measure effects of systemic senotherapies on cellular senescence, although this may not closely reflect changes in the lung (Hickson et al, 2019). Measurement of biomarkers in the blood has not been very useful in chronic lung diseases and circulating cells and mediators do not closely reflect their presence in lung tissue. However, because senescence may spread by EVs from the lungs via the circulation, it may be possible to isolate lung cell-derived EVs by their characteristic surface markers (Devulder et al, 2025). Senescent cells may also be monitored by positron emission tomography scanning. The radiotracer [^18^F]FPyGal to label SA-*β*-Gal in senescent cells has been used to detect senescent cells in osteoarthritis joints (Suryadevara et al, 2024). Imaging techniques may be important for assessing the effects of senotherapies in lung diseases in the future and would be suitable for long-term follow-up after senotherapy interventions. 3,4-Cyclohexenoesculetin *β*-d-galactopyranoside (S-Gal) has been used to detect SA-*β*-gal activity has been detected using magnetic resonance imaging and has the potential to measure senescence in peripheral tissues (Cui et al, 2010).

#### 2 Long-term administration

Many novel classes of senotherapy have been discovered using rapid throughput RNAi and CRISPR screens, but few of these drugs have been studied after long-term administration or in human cells (McHugh et al, 2025). Several classes of drug with antiaging effects are discussed in this review, and it is uncertain how these will compare in long-term administration and safety and how they will perform in different lung diseases, as their effects may be context-specific. Several drugs that are discussed are already approved for other disease indications and so may be repurposed as senotherapies. Although rapamycin and rapalogs reduce cellular senescence, there are concerns about long-term side effects and immunosuppression with these drugs. It is possible that lower doses of mTOR inhibitors that have less risk of adverse effects may be effective (Mannick et al, 2018). Metformin has several antiaging effects and is effective in animal models of chronic lung disease. The drug is well tolerated after chronic administration apart from gastrointestinal effects that are usually insufficient to lead to withdrawal (Dutta et al, 2023) Although there are no clinical trials of metformin as an antiaging drug in COPD, epidemiological studies have shown that metformin prescription in patients with COPD is associated with reduced mortality and slower disease progression (Yen et al, 2018; Mendy et al, 2019; Polverino et al, 2021). In order to reduce adverse effects of senotherapies it may be possible to discover combinations of drugs that act on different targets that additive or even synergistic effects on cellular senescence, but few studies have explored any combinations. The possibility of additive or synergistic effects of combining a senomorphic with a senolytic drug has not yet been explored, but elimination of senescent cells with a senolytic drug followed by a senomorphic drug may prevent the reaccumulation of senescent cells and therefore optimize therapy. Safety issues with senotherapies have been discussed above, but in addition to the potential adverse effects of senotherapies, it is likely that these treatments may be less effective in elderly patients due to the reduction in the capacity for regeneration in elderly patients. This suggests that the use of senotherapies early in the course of disease is likely to be more effective and that these drugs may be used prophylactically. Intermittent administration of senolytic drugs may abrogate any side effects, if side effects, such as thrombocytopenia with navitoclax, could be treated with platelet infusions.

#### 3 Lifestyle changes

In addition to drug therapies, lifestyle changes may be an important adjunct to complement senotherapies. Cigarette smoking and air pollution are major environmental factors that accelerate aging in chronic lung disease (Sin et al, 2023). Smoking cessation, clean cooking stoves, and avoidance of outdoor air pollution are therefore important strategies in reducing the burden of chronic respiratory diseases. Poor diets with increased fat and carbohydrates and a lack of dietary antioxidants and plant-derived substances, such as flavonoids (including quercetin and fisetin), are associated with worse lung function. Improved dietary intake of fresh fruit and vegetables, including a Mediterranean diet, may improve lung function in patients with COPD, although controlled trials of dietary interventions have not yet demonstrated reduced lung aging (Scoditti et al, 2019). Physical activity is also an important intervention, and exercise reduces systemic inflammation in elderly patients (Bautmans et al, 2021).

#### 4 Role of the microbiome

There is growing evidence that the microbiome may play an important role in aging diseases and dysbiosis is now recognized as one of the cardinal signs of aging (López-Otín et al, 2023). The gut microbiome changes with aging and may have an important influence on cellular senescence. Disturbance in the balance of microorganisms of the gut (dysbiosis) may lead to increased oxidative stress and the production of bacterial short-chain fatty acids that may promote senescence (Jang et al, 2024; Abavisani et al, 2025). For example, short-chain fatty acids secreted by bacteria may protect against the development of senescence though antioxidant effects and possible through activation of AMPK, although this has not been studied in lung cell senescence (Jang et al, 2024) The lung has a microbiome that may be altered in chronic lung diseases, such as COPD, IPF, CF, and asthma (Li et al, 2024). In COPD there is a profound dysbiosis of the lung microbiome, which is derived from the upper airways (Wang et al, 2018b). This is associated with chronic bacterial colonization of the lungs, with a predominance of *Haemophilus influenzae* and *Staphylococcus pneumoniae*. These bacteria drive further inflammation and an increased risk of exacerbations (Singh et al, 2014), but may also drive further senescence in the lung through increased oxidative stress as a result of increased neutrophil recruitment. Dysbiosis is also seen in IPF lungs (Spagnolo et al, 2019). Immunosenescence may itself predispose to lung colonization, and in COPD there is a defect in macrophage phagocytosis of bacteria, which correlates with the frequency of bacterial exacerbations (Singh et al, 2021). This suggests that normalizing the lung microbiome may have beneficial effects on cellular senescence in the lungs.

#### 5 Stem cell therapies

Effective senotherapies may allow recovery of stem cells and progenitors, including AT2 and Club cells to repair alveolar and airway damage. Mesenchymal stem cells (MSCs) have the potential for regenerating lung cells and reduce senescence in aged mouse lungs. MSCs promote alveolar regeneration in mice after lung injury, although it is difficult to retain these cells within the lungs (Iwatake et al, 2024). MSCs reverse bleomycin-induced pulmonary fibrosis in mice (Rubio et al, 2018). In cigarette smoke-exposed mice MSC and MSC-derived exosomes reduce lung inflammation and improve mitochondrial function (Maremanda et al, 2019). There is increasing interest in the potential for MSCs and MSC-derived EVs in the therapy of chronic lung diseases, although clinical studies to date have been disappointing (Hazrati et al, 2024).

#### 6 Heterogeneity of response

As discussed above, there is increasing evidence that senotherapies have different effects on different cell types, different stages of senescence, and in different diseases. Current senolytic therapies are relatively nonspecific but have differential effects according to the cause of senescence and the cell type involved. In the future more lineage-specific senolytics may be developed that target specific subtypes of senescent cell. This may involve linking the senotherapy to molecules that recognize specific cell markers. Further development of the CAR-T approach may lead to more specific senotherapies. In addition, the CAR approach may be applied to other cell types, such as NK cells and macrophages which may provide additional benefit. Single-cell RNA sequencing has highlighted the heterogeneity of senescent cells in different tissues and may provide novel biomarkers to distinguish different types of senescent cell in the future (Cohn et al, 2023). Machine-learning approaches may also be used to more accurately identify senescent cells based on morphological differences in nuclei and may detect heterogeneity of senescent cells in an unbiased manner (Duran et al, 2024).

## IX Conclusions

Cellular senescence is a major pathological mechanism in chronic lung diseases, including COPD, IPF, CF, bronchiectasis, severe neutrophilic asthma, and PAH, as well as COVID-19. Oxidative stress is an important driving mechanism through cigarette smoking and air pollution in COPD and from activated inflammatory cells in the lungs. All cell types in the lung may be susceptible to becoming senescent and accumulate in the lungs, which reduces capacity for repair, promotes fibrosis, and impacts the chronic inflammation in lung diseases. SASP mediators and EVs may spread senescence, resulting in disease progression. This suggests that targeting cellular senescence with senotherapies may be beneficial, and several drugs are in development based on in vitro studies and using animal models of chronic lung disease. Senomorphics inhibit the development of cellular senescence and their secretion of SASP mediators and EVs, which may reduce disease progression and comorbidities in patients with COPD. Several drugs target PI3K-mTOR signaling, sirtuins, and oxidative stress from repurposed molecules, such as metformin, rapamycin, and resveratrol, as well as novel drugs. Senolytic drugs eliminate senescent cells and may be effective as intermittent therapies. Several classes of senolytic drug have now been identified, including several Bcl-2/Bcl-X_L_ inhibitors, FOXO4-p53 inhibitors, HSP-90 inhibitors, and cardiac glycosides. Although senotherapeutics have been extensively investigated in animal models of aging, there are relatively few studies in models of chronic lung disease, apart from the bleomycin mouse model of IPF. More clinical studies are needed, either with repurposed drugs or with new drugs as they become available. There is uncertainty about which are the most useful biomarkers to demonstrate effects on cellular senescence in the lungs and it is not yet clear which clinical measurements are most appropriate. Measurements of lung function change slowly in chronic lung disease, but imaging techniques may be developed to assess drug interventions more rapidly. There is uncertainty about the long-term safety of senotherapies in relation to wound healing and cancer risk, but so far there do not seem to be significant safety issues in the few clinical studies reported. Senolytic therapies may be effective with intermittent administration so acute adverse effects may be managed. There is no doubt that senotherapies will become an important therapeutic approach for chronic lung disease and their comorbidities in the future and there is now a strong impetus to find novel effective therapies.

## Conflict of interest

PB has received research grant funding from UK Medical Research Council, Asthma Lung UK, AstraZeneca and Boehringer Ingelheim, and has participated in scientific advisory boards for AstraZeneca, EpiEndo, Novartis, and PulmoBioMed.
